# Indicators to identify cancer screening providers with suboptimal case detection: A scoping review

**DOI:** 10.1002/ijc.70264

**Published:** 2025-11-29

**Authors:** Jiayao Lei, Milena Falcaro, Adam R. Brentnall, James F. O'Mahony, Sisse Helle Njor, Matejka Rebolj

**Affiliations:** ^1^ Centre for Cancer Screening, Prevention, and Early Diagnosis, Wolfson Institute of Population Health Queen Mary University of London London UK; ^2^ Department of Medical Epidemiology and Biostatistics Karolinska Institutet Stockholm Sweden; ^3^ Centre for Evaluation and Methods, Wolfson Institute of Population Health Queen Mary University of London London UK; ^4^ School of Economics University College Dublin Dublin Ireland; ^5^ Department for Data, Innovation and Research Vejle Hospital, University Hospital of Southern Denmark Vejle Denmark; ^6^ Department of Regional Health Research University of Southern Denmark Odense Denmark; ^7^ Department of Clinical Medicine Aarhus University Aarhus Denmark

**Keywords:** cancer screening, evaluation, indicators, methods, sensitivity

## Abstract

Several international guidelines consider sensitivity (of test, episode, or programme) and related measures of the detection of prevalent cases of target disease to be among key performance indicators for quality control of routine cancer screening programmes and use them to identify suboptimal providers. We aimed to describe the variability encountered in real‐world settings around the measurement of these quantities in cervical and colorectal cancer screening, where the target for disease detection includes preinvasive disease. We performed a scoping review of grey literature, including international guidelines, annual statistical reports, and other official documents from European cervical and colorectal screening programmes. From the reviewed material, we extracted information on 20 measures used for this purpose. Some measures have been adopted in several programmes, but none have been used in all, not even within the same cancer type. While many of the methods might appear plausible for the intended use, our analysis showed that when applied to routinely collected data they may provide misleading or uninterpretable estimates of the ability of individual providers and the service as a whole to detect prevalent cases. Screening programmes should be cautious in their choice and interpretation of these measures. Further methodological development is required to better support policymakers and quality control managers in prioritising measures that are fit for purpose in routine cancer screening programmes.

## Graphical abstract

What's new?

Quality control of cancer screening programs is essential to maintaining high public health standards. Here, the authors reviewed monitoring of case detection in cervical and colorectal cancer screening programs in Europe, and evaluated its effectiveness. They analysed 20 different measures used for this purpose and showed that many of them provide false or misleading estimates of detection ability. They conclude that further methodological development is needed to improve performance monitoring of cancer screening programs.

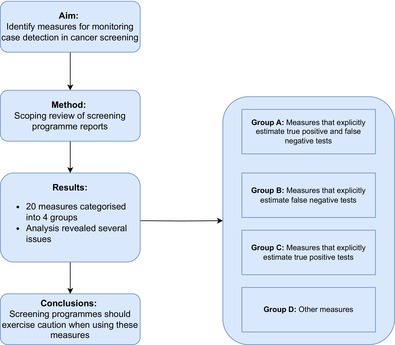

**Abbreviations**
CC: cervical cancer
CIN: cervical intraepithelial neoplasia
CRC: colorectal cancer
FIT: faecal immunochemical tests
FN: false‐negative tests
FP: false‐positive tests
gFOBT: guaiac‐based faecal occult blood tests
HPV: human papillomavirus
IARC: International Agency for Research on Cancer
inc: incidence
PCCRC: post‐colonoscopy colorectal cancer
PPV: positive predictive value
prev: prevalence
reatt: reattendance rate at the next screening round
SE_ref_
: sensitivity of the reference test
TN: true‐negative tests
TP: true‐positive tests

## 1 INTRODUCTION

Cancer screening programmes are massive health care undertakings that offer tests to millions of apparently healthy individuals every year. Quality control of screening processes is critical in identifying issues with the performance of the programme and individual providers. This helps to direct quality‐enhancing interventions where they are most needed and ensure that desired standards and targets are met.^1^, ^2^, ^3^ Measures that estimate the ability of providers to detect prevalent cases of target disease, including but not limited to test sensitivity, are considered by many to be key for quality control.^3^, ^4^, ^5^, ^6^, ^7^, ^8^

Sensitivity is not a fixed property of a screening test. It may vary depending on the disease spectrum,^9^, ^10^ that is, screening tests tend to be more highly sensitive for more developed disease. The disease spectrum observed in a population is, in turn, influenced by the population's case‐mix. In routine implementation, it may also vary between screening providers depending on their ability to recognise and interpret abnormalities. Some examples of screening tests relying on subjective interpretation include cytology in cervical screening, mammography in breast screening, and endoscopic methods in colorectal screening. Sensitivity may even differ between providers when they use what are considered to be “objective” screening tests such as faecal immunochemical (FIT) and human papillomavirus (HPV) tests; here, loss in test sensitivity may still occur due to variation in collection methods, laboratory handling and processing methods, or the assay (brand) used for the detection of target abnormalities. Recent real‐world examples from the Netherlands showed that test sensitivity varied both with differences between clinician and self‐collection when the HPV assay brand was the same^11^, ^12^ and with differences in HPV assay brands when the self‐collection device was the same.^11^, ^12^, ^13^

In principle, the best method to estimate “true” sensitivity would be to apply both the screening and an *accurate* reference test on everyone, and then determine how many of the lesions detected by the reference test are found by the screening test. Thus, “true” sensitivity is defined as the ratio of correctly identified individuals with the disease (true‐positive tests, TP) to the total number with the disease, i.e., the sum of true‐positive and false‐negative test results (false‐negative tests, FN); that is (TP)/(TP + FN) (Table 1). This measure is often referred to as the “detection” method of calculating screening sensitivity.^14^, ^15^

**TABLE 1:** Cross tabulation of screening test results against true disease status, where both outcomes are binary.

	Individuals with disease	Individuals without disease	Total
Screening test positive	True‐positive (TP)	False‐positive (FP)	TP + FP
Screening test negative	False‐negative (FN)	True‐negative (TN)	FN + TN
Total	TP + FN	FP + TN	N = TP + FP + TN + FN

Abbreviations: FN, false‐negative tests, the number of individuals with a negative screening test who have the target disease; FP, false‐positive tests, the number of individuals with a positive screening test who do not have the target disease; TN, true‐negative tests, the number of individuals with a negative screening test who do not have the target disease; TP, true‐positive tests, the number of individuals with a positive screening test who have the target disease.

Investigating all screened individuals with perfectly (or at least highly) accurate reference tests is sometimes possible in clinical trials. But such use of reference tests is typically impractical within population‐based service screening (Appendix A), and “true” test sensitivity must be estimated using routinely collected data. When the “detection” method is used in this context, TP cases are usually defined as asymptomatic cancers that were detected in individuals with abnormal screening tests and thereafter confirmed with *routine* reference tests. FN cases, on the other hand, are usually defined as those identified from the passive surveillance of interval cancers, that is, cancers in individuals with a negative screening test who presented with symptoms before the next screening round.^6^, ^16^ Both are imperfect approximations of the “true” TP and FN cases. Some of the “true” TP may not be recorded in routinely collected data because they were not verified, that is, some screen‐positive individuals did not undergo the reference test, or the reference test failed to identify a prevalent lesion. The group of cases labelled as interval cancers, on the other hand, may miss “true” FN cases where early signs of the abnormality are present at the time of screening but symptoms take longer to develop and/or be recognised; it can also erroneously include incident cases that only developed after screening and there was nothing to be detected in the screening test (i.e., de novo lesions). The distinction between “true” interval cancers and “true” incident cases is often impossible to make based on the routinely available data. Moreover, interval cancers can only be identified (several) years after the FN screening test. In practical terms, this means that quality‐enhancing interventions which rely on identification of FN tests through interval cancer registration can only be implemented after a considerable delay.

The complexity of measuring screening sensitivity in routine screening programmes further increases when the targets for detection include preinvasive disease.^7^ This is the case for cervical and colorectal cancers, where treatment of high‐grade cervical intraepithelial neoplasia (CIN2 and CIN3) and advanced adenoma, respectively, stops the progression to cancer. Both of these tend to be asymptomatic and can only be detected through screening; and while these lesions often progress very slowly, many never progress to cancer.^17^, ^18^

Given the challenges involved in directly estimating sensitivity, screening programmes and expert groups advising them have adopted various additional process indicators to measure the providers' ability to detect prevalent cases. As with sensitivity estimation, a difficulty with these process indicators lies in striking the balance between simplicity, timeliness, and informational value. The recommended measures are, with few exceptions, often based on methods developed primarily for analysis of clinical trials or for settings where the target for detection is invasive disease.^15^, ^19^, ^20^, ^21^

Some of these measures are reported in the programmes' annual statistical reports. Although such reports are typically published, they are often less readily accessible than searchable scientific collections.^3^ This may explain why measures of case detection—that help direct quality‐enhancing interventions to improve screening for millions of people—have received relatively little scrutiny, and motivated us to perform a scoping review of publicly available reports and other relevant documents from population‐based programmes across Europe. Our aims were: (i) to gain insight into the recent practice of monitoring the sensitivity and related measures of case detection in cervical and colorectal screening services; (ii) to describe the variability in the existing practices; (iii) to critically reflect on those practices; and (iv) to identify the areas in need of further methodological development.

## 2 METHODS

### 2.1 Search strategy

Our main goal was to identify measures of screening sensitivity and related measures of case detection in cervical and colorectal screening. We were interested in measures that are currently being used, were previously used, or have been proposed but not yet used. Previously used and proposed measures were included to offer a more comprehensive insight of the range of ideas considered, even if they were not used by screening services at the time of our review. We searched for electronic copies of documents written by the teams that manage or monitor, advise, or are otherwise associated with European cervical or colorectal screening programmes at national or supranational (e.g., International Agency for Research on Cancer [IARC] or similar) levels, which either (i) report the annual programme statistics or (ii) provide a methodological plan for the said statistics. We limited our searches to European countries, as defined in a major report of cervical screening worldwide^22^; this included 43 out of 53 countries within the World Health Organisation's European region, whereas the remaining 10 countries were categorised under Asia.

No central repository exists for these documents. Our prior experience indicated that many of these documents would be available online, usually in a local language. We also expected that the availability of documents would differ between programmes, that is, some may be clearly and consistently published online, others less so. The anticipated inconsistency in making the documentation available for the public was expected to limit the utility of a common search strategy for all countries. Hence, we undertook a two‐stage non‐systematic search using results from the internet search engine Google, searched from the UK, Denmark, or Sweden. In the first stage, we identified countries or regions with organised cervical^22^ and/or colorectal screening programmes (defined as a centralised service relying on individual‐level invitations at pre‐specified ages and/or intervals) and an online presence thereof. Countries without organised screening were omitted from the searches. In the second stage, we examined the programmes' websites and used supplementary Google searches in English and local languages to help identify the potentially relevant documents. All searches were undertaken between April 2022 and April 2023, without restriction on the publication year or the language. The searches were operated with the help of an online translation tool into English (https://translate.google.com/) where necessary, but at least one of JL, SHN, and MR could read the documents in the original language other than English when they were available in Danish, Swedish, Norwegian, Dutch (including Flemish), German, Slovenian, or Bosnian (including Croatian, Serbian, and Montenegrin). We also reviewed the reports' reference lists and perused our own reference archives for further reports of relevance. The final list of the resources identified from the 43 countries is available in Data S1, Supporting Information.

### 2.2 Selection of resources

All identified resources were scanned for any mention of sensitivity or other measures of detection of cases that are prevalent at the time of screening, related to the screening test, the diagnostic process, or similar. We used keywords: “sensitivity,” “interval cancer,” “detection,” “accuracy,” “reliability,” “performance,” “effectiveness,” or “quality.” Once a relevant performance measure was identified, we extracted the definition, if available, including the numerator, denominator, and any additional explanation such as the inclusion and exclusion criteria or the lookout periods (i.e., the period from a screening event such as the completion of a screening test to a relevant diagnostic outcome such as a diagnosis of an interval cancer). To document the justification for inclusion in our review, we also recorded the full text interpretation of the calculated values. If none of the keywords were found, we excluded the report from further consideration. The results of this process, including the retained and the excluded reports, were recorded for each cancer site separately.

The searches and selection of the resources were initially undertaken by a single researcher (MR for cervical and JL for colorectal cancer). A second researcher performed checks for completeness (extraction of the information from the reports, with an independent selection of resources for several countries) and accuracy (JL for cervical and SHN for colorectal cancer). SHN (cervical) and MR (colorectal) acted as arbiters if necessary.

### 2.3 Data charting

The wide selection of search keywords helped us examine how variable the practice and ideas underlying the measurements of sensitivity or other measures of case detection in routine screening programmes have been. We considered a performance measure eligible for our study if at least one of the programme reports interpreted it as (at least partially) indicative of the ability of a screening provider or service as a whole to detect the target disease. At this stage, we were not prescriptive even though some of the measures that were identified in this way could only be interpreted as indirectly related to screening sensitivity (this point may or may not have been addressed by the authors of the programme reports).

Categorisation of the different measures was not done a priori because understanding their variability was one of the aims of this review. Once all searches were completed, the identified measures were grouped depending on whether they explicitly estimate the numbers of individuals with both TP and FN tests (Group A) or just one of them (Group B: FN only, Group C: TP only; see Table 2, using definitions from Table 1). Measures using a different approach were grouped separately (Group D). Within each group, measures were further classified depending on the subgroups of the screened population whose data enter the calculations or whether histological confirmation is required for their calculation. The data charting process was undertaken by one researcher (MR) and was checked by two others (JL, SHN).

**TABLE 2:** Measures from European cervical and colorectal screening programmes addressing the services' ability to detect cases of prevalent disease.

Measure #	Functional form (fraction)		Cervical screening^a^		Colorectal screening^b^	
	Numerator (number of individuals with…)	Denominator (number of individuals with…)	Recommended measures	Measures with reported data	Recommended measures	Measures with reported data
Group A: Measures that explicitly enumerate both true‐positive and false‐negative tests						
#1	Screen‐detected cancers	Screen‐detected cancers + interval cancers		1		3
#2	Screen‐detected lesions	Screen‐detected lesions + interval cancer + screen‐detected lesions at the subsequent round in initially screen‐negative individuals		1		
#3	Screen‐detected lesions	Screen‐detected lesions + interval cancer	1			
#4	Abnormal screening tests identified at initial evaluation	Abnormal screening tests identified at initial evaluation + initially negative screening tests with results upgraded to abnormal at rapid review		1		
#5	Screen‐detected cancers + post‐reference test cancers + screen‐positive cancers that were lost to follow‐up	Screen‐detected cancers + post‐reference test cancers + screen‐positive cancers that were lost to follow‐up + interval cancers				1
#6	Screen‐detected preinvasive lesions + preinvasive lesions detected after a negative reference test + preinvasive lesions diagnosed in screen‐positive individuals who did not undergo reference testing	Screen‐detected preinvasive lesions + preinvasive lesions detected after a negative reference test + preinvasive lesions diagnosed in screen‐positive individuals who did not undergo reference testing + preinvasive lesions in screen‐negative individuals detected during the interval				1
#7	Cancers after a positive screen and a negative reference test	Cancers after a positive screen and a negative reference test + screen‐detected cancers				1
#8	Cancers with recent negative screening tests in their screening history	All cancers diagnosed in a specific period (screen‐detected or symptomatic)	4	8		
#9	Interval cancers	Expected incidence of (the target) cancer, adjusted for non‐participation^c^			1	
Group B: Measures that explicitly enumerate false‐negative tests						
#10	Interval cancers (after negative screening tests)	Negative screens (or person‐years after a negative screen)	4	1	2	2
#11	Lesions (after negative screening tests)	Negative screens	1	3		
#12	Interval cancers (after negative screening tests or after positive screening tests followed by negative reference tests)	Negative screens + positive screens followed by negative reference tests	1		1	
#13	Cancers diagnosed after positive screening tests followed by negative reference tests	Negative reference tests				1
#14	Interval cancers (after negative screening tests or after positive screening tests followed by negative reference tests)	All screens			1	
#15	Cancers diagnoses after positive screening tests followed by negative reference tests	Reference tests				1
Group C: Measures that explicitly enumerate true‐positive tests						
#16	Screen‐detected lesions	Screening tests	2	5	6	7
#17	Screen‐detected lesions	(none)		1		
#18	Screen‐detected lesions	Reference tests			5	4
Group D: Other measures						
#19	Referred to reference test	Screening tests	1			
#20	Low‐grade abnormal screening test	High(er)‐grade abnormal screening test		1		

*Note*: Counts refer to the numbers of screening programmes that have used the specific measures. In TP, FN, and related categories, “cancers” refers to cases of target cancer (either cervical or colorectal cancer), “lesions” refers to preinvasive lesions or cancer (either CIN2+, CIN3+ for cervical cancer, or advanced adenoma or worse for colorectal cancer), whereas “preinvasive lesions” exclude cancers. Reference tests are diagnostic tests used to investigate individuals with positive screening tests (usually colposcopy in cervical screening and colonoscopy in colorectal screening). The counts of TP and FN tests would be obtained from real‐world, routine registration of data within the screening programme. Misclassification owing to missing data (e.g., missing reference standard due to loss to follow‐up, emigration, deaths, etc.), incomplete verification (as in, e.g., the counting of interval cancers with an early cut‐off for the lookout period), and other factors can affect these counts.

Abbreviations: CC, cervical cancer; CRC, colorectal cancer.

^a^ References for measures in cervical screening. For countries: Belgium (Flanders),^74^, ^75^ Denmark,^76^ Finland,^77^ Germany,^78^ Ireland,^79^, ^80^ Italy,^81^, ^82^, ^83^ Netherlands,^84^, ^85^ Norway,^86^ Slovenia,^87^, ^88^, ^89^ Sweden,^90^, ^91^, ^92^, ^93^ UK (England),^94^, ^95^, ^96^, ^97^ UK (Northern Ireland),^98^ UK (Scotland),^99^ UK (Wales).^100^ From international groups: Coleman et al.,^19^ IARC,^5^ Arbyn et al.^20^ Measures for the four UK countries were counted separately, as each country implemented their own screening programmes.

^b^ References for measures in colorectal screening. For countries: Belgium (Flanders),^101^ Belgium (Wallonia),^102^ Denmark,^103^, ^104^, ^105^ Finland,^106^ France,^107^ Germany,^108^ Ireland,^109^, ^110^, ^111^ Italy,^112^, ^113^, ^114^ Luxembourg,^115^ Netherlands,^116^ Norway,^117^ Serbia,^118^ Slovenia,^119^ Spain (Basque Country),^120^ Sweden,^121^, ^122^ UK (England),^123^, ^124^ UK (Northern Ireland),^125^ UK (Scotland),^126^ UK (Wales).^127^ From international groups: Segnan et al.^128^ Measures for the four UK countries and the two Belgian regions were counted separately, as each implemented their own screening programmes.

^c^ Calculated as [pre‐screening incidence of cancer − (1 − uptake) × incidence of cancer among non‐participants]/uptake, recommended to be standardised for age and sex.

We counted how many screening programmes considered each measure. We stratified these counts by cancer type and whether the identified reports included any data to inform calculations following the method underlying the specific measures.

We then considered whether there were any limitations that might prevent a measure from reliably estimating the “true” ability to detect prevalent cases or from enabling fair comparisons between health care providers, when using routinely collected data. These considerations were based on the functional forms of the measures (equations). Hypothetical examples were used to illustrate selected issues.

## 3 RESULTS AND CRITICAL REFLECTION ON THE MEASURES

For cervical screening, we identified measures of case detection from 11 countries and three international groups; for colorectal screening, this was 15 countries and one international group. In total, these sources considered 20 unique measures. These measures related to (i) the detection of cases by the screening or reference test alone (“test sensitivity”), (ii) the detection by the screening test, any triage, and the resultant reference tests combined (“episode sensitivity”), and (iii) the detection following an invitation to screening, where disease may remain undetected because of issues with the screening, triage, or reference tests, or because of failure to engage parts of the target population (“programme sensitivity”).^23^ Table 2 provides a summary list of the measures and their frequency of use. Although some measures were considered in several programmes, none were considered across all programmes. Only a few reports discussed motivation for the inclusion of the specific measures, their validation, or potential caveats.

### 3.1 Group A: Measures that explicitly enumerate both TP and FN tests

These measures seek to estimate both TP and FN tests. Most of them evaluate sensitivity using variations of the “detection” method, defined as TP/(TP + FN), but alternative estimators have also been proposed.

The first approach (measure #1) is a direct application of the “detection method” to estimate sensitivity. It defines TP tests as cervical or colorectal cancers diagnosed at colposcopy or colonoscopy after a positive screening test, and FN cases as interval cancers presenting after a negative screening test. This measure has already been critiqued by others.^6^, ^16^ Briefly, as explained above, this measure does not allow for quality control in real‐time. It incorporates differential verification of the lesions in the numerator and the denominator. Diagnostic verification is offered systematically to all asymptomatic screen‐positive individuals, meaning that screen‐detected cases can be diagnosed fairly shortly after screening. Conversely, the timing of interval cancer diagnosis, which may affect whether these cases are included in the calculations at all, depends on the affected individual's recognition of symptoms and the waiting time for a reference test (e.g., colonoscopy) appointment.^16^, ^24^, ^25^, ^26^, ^27^, ^28^, ^29^, ^30^ Additionally, an implicit assumption is that symptomatic cancers were present at screening but missed. Hence, with longer lookout periods the “detection” method may misclassify truly incident (de novo) cases as FN on the screening test; but may, with shorter lookout periods, also fail to enumerate truly missed FN cases with longer preclinical phases that take longer to become symptomatic.^6^, ^16^, ^25^

Another issue with measure #1 (and other measures estimating TP tests) is the accuracy and completeness of the gold‐standard reference test. In practice, the accuracy of the reference test may vary between providers.^31^, ^32^, ^33^ Moreover, some TP disease may be missed when screen‐positive patients do not receive the reference test, for example, because patients did not obtain the recommended clinical management. Indeed, differences in the utilisation of reference testing have been observed even in countries with free access to health care, uniform national health care systems, and where national screening and follow‐up guidelines exist.^34^, ^35^, ^36^ Finally, we note that the “detection” method does not address the detection of preinvasive lesions and hence does not provide an opportunity for programmes to monitor the extent to which their services contribute to the prevention of cancer.

Some alternative measures related to the “detection” method have also been considered. Measure #2 has the same functional form as #1, but defines the target disease outcome as preinvasive disease or worse (such as CIN2/3 and cancer combined). As with measure #1, TP tests are found using reference testing among screen‐positive individuals. Acknowledging that, unlike cancer, preinvasive disease is asymptomatic and only detectable through screening, FN tests may include TP cases from the next screening round in those who tested negative at the previous round (note, interval cancers may also be added to this number).

Potential issues with the interpretation of estimates using method #2 include those illustrated with hypothetical scenarios in Appendix B. They show that this approach will give favourable appraisals to under‐performing providers as they will typically generate FN tests less often; that is, TP tests at the subsequent round are less likely because “true” sensitivity is lower. Furthermore, these estimates can be positively or negatively affected by other factors that providers have no control over: (i) where the case‐mix at the subsequent screening round includes a higher proportion of de novo lesions, which inflates the numbers of FN tests and may be particularly punishing for, e.g., cervical screening providers serving younger populations^37^ and those that are performing well; (ii) where FN tests cannot be enumerated because the individuals no longer engage with the screening programme, the proportion of whom can differ between catchment areas^38^; or (iii) where the numbers of TP tests are underestimated because reference tests do not achieve a high level of diagnostic sensitivity. Consequently, the screening sensitivity estimated using this method may differ substantially from the “true” sensitivity. It is impossible to determine the direction of the bias from routinely collected data and the measure therefore cannot be recommended.

Measure #3 is another direct modification of #1 and defines TP tests as screen‐detected preinvasive disease or worse and FN tests as interval cancers. This measure is affected by a numerical imbalance between the detected TP and missed FN lesions. For example, in cytology and HPV‐based cervical screening undertaken in well‐screened populations the detection of CIN2/3 is often >100 times higher than the number of interval cancers.^39^, ^40^ Consequently, the estimates on this measure will be close to 100%, even if “true” sensitivity is much less than 100%. This is expected when progression from preinvasive lesions to invasive disease is relatively slow, as is the case for cervical cancer.^18^, ^41^, ^42^ Issues are illustrated in Appendix C in more detail, showing the difficulties in distinguishing between poorly and well‐performing providers.

Measure #4 is a variant of #1 that has been considered in cytology‐based cervical screening. It uses a two‐stage slide evaluation process, so that a rapid review of samples deemed normal at initial evaluation provides a safety net for cases missed by the initial evaluation. Here, TP tests are those where abnormalities are found at the initial evaluation of cytological slides, whereas FN tests are those where abnormalities are recognised only at rapid review. Where robust certification procedures are in place for rapid reviewers, this approach could provide a reasonable early warning signal to indicate problems with the sensitivity of specific screeners. Nevertheless, rapid review of cytology is an unverified reference test: like the original evaluation, it relies on a subjective interpretation of changes, its quality depends on the reviewer's expertise, and its outcomes are not verified with histology.

The definition of TP screen‐detected cases following #1 is sometimes extended to include cancers that were identified by the screening test but were (initially) not correctly verified by the reference test (measure #5). The latter may include cases in screen‐positive individuals who did not present for the reference test, or those diagnosed at the second or later reference test. In colorectal screening, these cases are often called post‐colonoscopy colorectal cancers (PCCRC) and are diagnosed with surveillance colonoscopy or following clinical symptoms, after the initial colonoscopy failed to identify lesions worse than adenomas or any lesions at all. Measure #6 is a variant of measure #5, where the TP definition includes asymptomatic preinvasive lesions (e.g., adenoma) but excludes cancers (#6). The inclusion of preinvasive lesions raises some of the same concerns as described for #2. The application of this measure in colorectal screening, however, does not include TP tests at the next screening round in the definition of FN. Nevertheless, whether preinvasive lesions are detected during the screening interval is likely to depend (to an extent) on self‐selection among screen‐negative individuals, among whom reference testing is in principle not expected. This self‐selection may differ between populations served by different providers and be associated with various factors such as worry or opportunity. Consequently, some of the elements needed to calculate #6 may differ between providers for reasons unrelated to “true” sensitivity.

Measure #7 aims to address issues with the sensitivity of reference testing. The measure is defined as the proportion of cancers after a negative reference test which were test‐positive at primary screening (e.g., PCCRC), out of all cancers with a positive primary screening test. Here, PCCRC cases are included in the FN definition, unlike for #5, because #5 and #7 are used to evaluate different aspects of sensitivity in the screening process.

Measure #8 is a relatively common measure addressing gaps in timely case detection for cervical screening. These gaps can be due to recent negative screening tests, various failures in the follow‐up of abnormalities, or non‐attendance. To obtain this measure, historical records are consulted for each cancer case to determine, if appropriate, the most likely part of the screening process that failed. The evaluation of screening histories is often done using cancer audits.^43^, ^44^ Some cervical screening audits mandate re‐examination of the screening test, such as by reviewing a patient's historical cytology screening slides to better determine whether the cancer was preceded by a truly FN test or a fast‐growing de novo case; this re‐examination is, of course, informed by the knowledge of the existing cancer diagnosis. A further extension of this approach in some programmes compares screening histories of cases with a sample from the population without cervical cancer (controls). When comparing screening histories between cases and controls, temporal changes in the chance of being diagnosed with cancer after a negative screen may be related to changes in screening sensitivity.^45^, ^46^, ^47^ Measure #8 shares some of the weaknesses with those approaches that rely on interval cancer rates, in that it uses a retrospective definition of a screening failure to evaluate test sensitivity. It is also prone to variation in the quality of the screening history review when the reviews are undertaken locally by each provider separately. Moreover, access to complete data on the woman's screening history including that outside of the hospital where cancer was diagnosed is important, but not always available. In this circumstance it is possible to misclassify some as having not attended screening previously, rather than having a false‐negative test or another failure in the screening process.^48^

Finally, measure #9 has been recommended for new programmes in previously unscreened populations. This defines lack of sensitivity as the ratio of interval cancer incidence, to the expected cancer incidence without screening (i.e., the incidence rate that would be expected in the screened population had screening not begun), after adjustment for differences in the population who participate or not in screening. Risk factors for cancer and the background incidence in the screening cohorts change over time,^49^ making pre‐screening incidence less reliable for expected background incidence as time goes on.^50^ Hence, this measure typically ceases to be informative in the established screening programmes, although it continues to be used occasionally.^51^, ^52^

### 3.2 Group B: Measures that explicitly enumerate FN tests

The measures in this group focus on the frequency of FN tests, usually defined as interval cancer incidence or, sometimes, a presumed equivalent in preinvasive lesions. The rationale is that low values are consistent with low numbers of FN tests and high screening sensitivity, all else being equal.

Measure #10 is the most common from this group. It is often considered a key quality assurance indicator by policy makers, researchers, and programme managers.^5^, ^19^, ^20^, ^21^, ^53^, ^54^ Here, the number of interval cancers is compared to the number of individuals with, or person‐years after, negative screening tests, in the form of FN/(FN + TN) (Table 1). This is the complement of the negative predictive value of a screening test, TN/(FN + TN). Interval cancers are counted either only during the interval between screens (when fast‐progressing cancers are more likely represented than at screening), or during the interval and at the next screening test combined, or only at the next screening test; though this is often left undefined and only the lookout period may be specified. As explained above, interval cancers are diagnosed depending on the timely recognition of symptoms^24^; and simple counts retrieved from routinely collected data are at risk of bias due to over‐ or under‐estimation.^6^, ^16^, ^25^ Furthermore, variation in the true background risk^54^ may affect the incidence of interval cancers and thus mask or exaggerate true differences between screening providers or between time periods (Appendix D). In practical applications in cervical screening, for example, interval cancer rates are expected to vary between the different providers with the availability and uptake of HPV vaccination even when the quality of their screening service is equal.^55^ Hence, caution is required when this measure is used to compare individual providers or interpret temporal changes within the screening service. Finally, measure #10 and any other measures relying on the identification of interval cancers may be unstable due to a typically low frequency of interval cancers among screen‐negative individuals. Instability may be less of an issue when monitoring the performance of larger screening units or the screening service as a whole.^56^

Measure #11 has been used for cervical screening by extending #10 to include high‐grade preinvasive lesions (CIN2/3) and cancers, that is, CIN2+. When only preinvasive lesions that surface before the next screening round are included in the counts of apparent FN tests, the interpretation of the results is unclear. Incidental diagnoses of high‐grade CIN may be made in self‐selected subgroups and undercount the true number of missed lesions. When lesions detected at the subsequent screening round are included, similar issues arise as discussed for measure #2.

Other measures relying on FN cases alone have been proposed to help identify issues with disease detection, for example, by including issues with the accuracy of the reference test. For this purpose, measure #12 defines FN cases to include interval cancers after a negative screening test, and those after a negative reference test in screen‐positive individuals such as PCCRC (compare with #5, where the purpose is to estimate screening test sensitivity and so reference‐negative cases are considered TP screen‐detected cases). Following the recent recommendation,^8^, ^44^ it is likely that measure #12 will be used more widely to monitor cervical screening in the future. A caveat with measure #12 is that a well‐performing screening test process may mask issues with poorly performing reference testing or vice versa. Without breaking the evaluation measure down into separate components, it may be difficult to identify which part of the screening service requires improvement. This is done by measure #13, which only considers negative reference tests following positive primary screens and the subsequent cancers. Finally, measures #14 and #15 are similar to measures #12 and #13, respectively, but include all individuals with completed screening tests (#14) or reference tests (#15). The inflated denominators in these two measures, including people with positive tests who (by definition) do not contribute cases arising after negative tests, may conceal some of the problems with case detection. The extent of this problem depends on the case mix in the population (which may differ between providers and would at minimum need to be adjusted for) but will be particularly prominent among the referred populations who were pre‐selected to undergo reference testing to investigate their elevated risk of underlying lesions, evidenced by positive screening and triage test results.

### 3.3 Group C: Measures that explicitly enumerate TP tests

Measure #16 is the most frequently used measure from this group. It compares the numbers of TP tests defined as detected lesions, whether preinvasive or worse or cancers alone, with the number of screened individuals. It can be expressed as TP/(TP + FP + TN + FN) (Table 1). In principle, high values of #16 can be consistent with high screening sensitivity, all else being equal. Programmes reporting this measure tend to be cautious in its interpretation as a measure of test or episode sensitivity. This is because partial verification without attempting to estimate the number of FN tests may give an inflated impression of test sensitivity.^57^, ^58^ Furthermore, test sensitivity is only one factor contributing to disease detection, and potential confounders tend not to be adjusted for. Using a hypothetical example, Appendix E illustrates, for example, how a provider achieving lower “true” sensitivity can detect more target lesions in a higher‐risk population than a provider achieving high “true” sensitivity in a lower‐risk population. Our hypothetical example assumed a doubling of the risk between the “average” and “high” risk populations; this risk differential is not entirely without real‐world parallels as, for example, the prevalence of HPV infections and CIN2+ lesions may be more than 10 times greater in younger vs. older women.^39^ As another practical example demonstrating difficulty in identifying poorly performing providers from detection rates alone, we can also consider the complexities caused by the recent co‐occurring changes in cervical screening. Here, an increasing proportion of screened women have been vaccinated against HPV (which will decrease the detection rate)^59^; HPV testing is replacing cytology (which will increase the detection rate, particularly in the initial rounds)^40^; samples collected by clinicians are increasingly replaced by samples collected by women themselves (which will keep the detection rate at the same level or decrease it)^11^; the latter change may also increase the number of under‐screened women among those who participate (which may increase the detection rate).^11^ Because of the differences in women's screening behaviour, the combined impact of these changes may differ between screening providers even within the same year; and the impact will differ from one year to the next. In that case, at what point can quality managers be confident that a decreased detection rate is due to vaccination, rather than to issues with the detection of cases? Without more in‐depth analyses (which are usually not part of routine programme monitoring), measure #16 is not informative.

In some reports, this measure is further simplified by removing the denominator, that is, reporting only the numerator (#17). The interpretation of this simplified measure is unclear, as changes in population size and screening participation alter the number of detected lesions irrespective of a screening test's “true” sensitivity. We potentially see some rationale for using this measure in very specific circumstances such as assessing the effect of short‐term service closure due to the COVID‐19 pandemic. However, this application addresses the (potentially catastrophic) issues with abrupt decreases in screening participation and falls outside of the routine monitoring of the providers' service quality.

In colorectal screening the number of screen‐detected lesions in the numerator has been frequently compared with the number of individuals undergoing a reference test (colonoscopy) in the denominator (#18). Using notation from Table 1, this measure can take two functional forms. One is TP/(TP + FP + TN + FN) whenever colonoscopy is the primary screening test and is essentially equivalent to #16. In settings where colonoscopy is only used for diagnostic investigation of individuals with positive stool tests, the second form is TP/(TP + FP) and the TP and FP counts refer to the individuals undergoing the reference rather than the screening test. Here, this measure primarily helps monitor the quality of diagnostic services, with the rationale derived from studies that linked detection rates to longitudinal outcomes such as interval cancers and colorectal cancer mortality.^60^, ^61^ Some screening programmes set a minimum threshold that each colonoscopist is expected to achieve on this measure, though attempts to account for factors affecting the detection such as age, sex, or previous screening history^61^, ^62^, ^63^ are usually not described (and likely not made). When these factors are not accounted for, then at least in theory, colonoscopists exceeding the minimum threshold in a higher‐risk population may still provide a lower‐quality service than those barely or not reaching the threshold in a lower‐risk population. It is interesting to note that this is not how this measure is used in cervical screening; there, it is interpreted as a positive predictive value (PPV) of a colposcopy referral and usually reserved as an efficiency rather than a sensitivity measure (and hence could not be included in Table 2 for cervical screening). Nevertheless, real‐world examples from cervical screening underscore the importance of accounting for the population case‐mix in a measure like #18. Data from England have shown a temporal decline in the PPV for CIN2+ following the introduction of population‐based screening for the first cohorts vaccinated against HPV. The steady decline in the PPV as the proportion of vaccinated women increased (i.e., the case‐mix changed) was seen even though screening samples were collected by the same clinicians, processed in the same laboratories using identical HPV assays, were interpreted by the same cytology teams, and women with abnormalities were followed up in the same colposcopy units.^59^

### 3.4 Group D: Other measures

Examples from cervical screening that could not be categorised in Groups A–C include the proportion of screened individuals who are referred to colposcopy (#19, (TP + FP)/(TP + FP + TN + FN) using notation from Table 1) and the proportion of individuals with low‐grade abnormalities on the screening test compared to the numbers with high‐grade abnormalities (#20). The main premise behind these two measures is that sufficiently low values (for which the thresholds were likely set based on external data sources, though these tend not to be mentioned) may suggest that cases with the target disease are being missed by the screening test. Neither of these measures requires that screening test outcomes are validated against a clinical diagnosis and so are difficult to interpret in the absence of information on other process indicators (which include screening specificity and related measures).

## 4 DISCUSSION

As is evident from our review, in particular Table 2, there is a lack of consensus on measures addressing the accuracy of detection of prevalent cases of target disease used across European cervical and colorectal screening services. Ostensibly, many of these measures may seem appropriate for their intended use. Nevertheless, when applied to routinely collected screening data several measures provide empirical estimates that are difficult to interpret, and even potentially misleading. Both observations—the wide variety of appraisal metrics and potential issues with their validity—indicate that further methodological guidance and validation are urgently needed.

We recognise that screening programmes are often under pressure to provide evidence of service quality, and that to date there has been little robust guidance regarding performance measures. Methodological progress could contribute to more confident quality control, directing corrective action to where it is needed, avoiding staff frustration in mischaracterised units, and difficulties with retaining the well‐performing workforce. For now, policymakers and quality control managers are advised caution when selecting and interpreting their quality control measures.

Our analysis is distinct from previous reviews^3^, ^7^, ^53^, ^54^, ^64^ as it identified a larger number of sensitivity measures used, or considered for use, in European colorectal or cervical cancer screening programmes, and by challenging their suitability for this purpose. The latter was supported by hypothetical examples that illustrate the biases that these measures can produce when applied to routine screening data. The key to achieving this was focusing our review on grey literature. Nevertheless, some limitations ought to be acknowledged. A protocol for this scoping review was not registered, and we did not contact individual programmes directly to clarify details. Due to the required workload, we restricted our review to European regions. Further work should also discuss the definitions of the components that are entered into the calculations. These include separating primary screening samples from samples that are taken for other purposes (this distinction is relevant because of the likely spectrum effects),^9^, ^10^ adjusting for temporal and geographical variability of the populations served by different parts of the screening service (i.e., the case‐mix),^61^, ^63^ or deciding on the length of the lookout period to establish an outcome.^6^, ^16^ While these decisions do not affect the equations (the primary focus of our review), they affect the estimates. We note that this type of information is not reported consistently in the collected screening programme documentation, although it is probably included in other types of material such as internal notes. Finally, we acknowledge that our list of sensitivity measures may not be exhaustive and may miss some indicators used only in the less easily accessible statistical reports.

The complexity of evaluating suboptimal case detection in a routine cancer screening service is recognised.^6^, ^23^, ^65^ “Ideal” measures would be related to long‐term outcomes relevant for screening such as a reduction in cancer‐specific incidence, mortality or morbidity. These were described in cancer screening trials and long‐term service data^2^, ^61^; but such evidence is currently lacking in specific emerging contexts such as cervical screening of women vaccinated against HPV. A gap in the availability of interventional or observational data suitable for validation could be bridged by employing other more rigorous methods whose performance could be evaluated via statistical simulations. Other key characteristics of “ideal” measures for use in routine screening services are that they should be relatively easy to implement and provide rapid feedback.^3^, ^7^, ^53^ Simulation studies could help select and study the properties of measures that provide rapid feedback but are strongly related to long‐term outcomes. This work remains to be done. Even for quality control purposes within public health interventions as massive as cancer screening, however, services may face obstacles such as incomplete data registration, inability to link data from several sources, or inaccessibility due to increasingly restrictive privacy protections. With this in mind, it is not surprising that, as reported in Table 2, relatively simple even if unspecific measures such as those from group C (e.g., the detection rate) appear to be among the most popular across both cervical and colorectal screening programmes. Collaboration with those responsible for quality assurance and data access will be essential for identifying better indicators that are also feasible to monitor with the data available to the programmes.

In general, methods to address gaps in the sensitivity of diagnostic tests in symptomatic individuals cannot be directly applied to cancer screening. Meaningful translation to the screening context needs to consider factors such as detection of preinvasive lesions, asymptomatic presentation, and a low prevalence of cases in the examined population which underpins the often conservative clinical management pathways^25^, ^66^; dependence of a final diagnosis on the quality of a chain of testing stages^25^, ^30^; missing (often not‐at‐random) outcomes of the reference tests and delays in their application, which is in line with the established management pathways^27^, ^28^; imperfect routine reference tests; imperfect data sources; and a small size of some of the provider units.^56^ Some of these factors have been considered when discussing the caveats of the standard form of the “detection” method (measure #1).^26^, ^28^, ^67^ Our examination of potential limitations shows that similar methodological scrutiny would be welcome for other measures in Table 2.

A specific area requiring methodological development is monitoring the accuracy of processes downstream from a positive screening test such as triage, early recall, and diagnostic testing, and whose performance may vary substantially between the individual providers.^36^, ^68^, ^69^, ^70^, ^71^, ^72^ This is not only to provide a suitable set of measures to monitor the quality of those services in their own right, but also to separate issues that are related to diagnostic tests from those associated with screening tests. Except in a handful of colorectal screening programmes (Table 2), most screening programmes do not appear to monitor the quality of their diagnostic services systematically. The need to monitor the quality of screening processes from beginning (invitations, screening test) to end (clinical management, diagnosis, treatment, and test of cure) has been underscored by, for example, the global initiative to eliminate cervical cancer.^73^

In conclusion, this scoping review has sought to better support screening services and clarify various issues that they may encounter when they employ a range of available measures to monitor performance. International collaboration between scientists, clinicians, data access specialists, and programme staff is necessary for further methodological development to establish a set of service sensitivity measures that are fit for purpose in ongoing cancer screening programmes.

## AUTHOR CONTRIBUTIONS

**Jiayao Lei:** Data curation; investigation; validation; formal analysis; project administration; writing – original draft; writing – review and editing. **Milena Falcaro:** Writing – review and editing. **Adam R. Brentnall:** Writing – review and editing. **James F. O'Mahony:** Writing – review and editing. **Sisse Helle Njor:** Conceptualization; methodology; investigation; data curation; validation; formal analysis; supervision; writing – original draft; writing – review and editing; project administration. **Matejka Rebolj:** Conceptualization; methodology; investigation; data curation; validation; formal analysis; supervision; writing – original draft; writing – review and editing; project administration.

## FUNDING INFORMATION

Jiayao Lei: Swedish Research Council (references: 2021‐00289, 2023‐01809) and Swedish Cancer Society (No. 24 3862). Milena Falcaro and Matejka Rebolj: Cancer Research UK (reference: C8162/A29083). Adam R. Brentnall and Sisse Helle Njor: No specific funding. James F. O'Mahony: University College Dublin Ad Astra programme.

## CONFLICT OF INTEREST STATEMENT

Jiayao Lei, Milena Falcaro, Adam R. Brentnall, James F. O'Mahony: No conflicts of interest to declare. Sisse Helle Njor: Employed as epidemiologist at the Danish Clinical Quality Program (RKKP) during 2015–2022; and was epidemiologist for the Danish Colorectal Cancer Screening Database and the Danish Quality Database for Mammography Screening. Matejka Rebolj: Public Health England and the UK National Screening Committee provided funding for the epidemiological evaluation of various cervical screening studies; current and former member of various expert groups providing advice to NHS England cervical screening programme; attended meetings with HPV test manufacturers; fees for attendance at advisory board and other meetings organised by Hologic, shared with employer, including travel cost reimbursement if applicable.

## Supporting information


**Data S1.** Supporting Information.

## Figures and Tables

**FIGURE A1:**
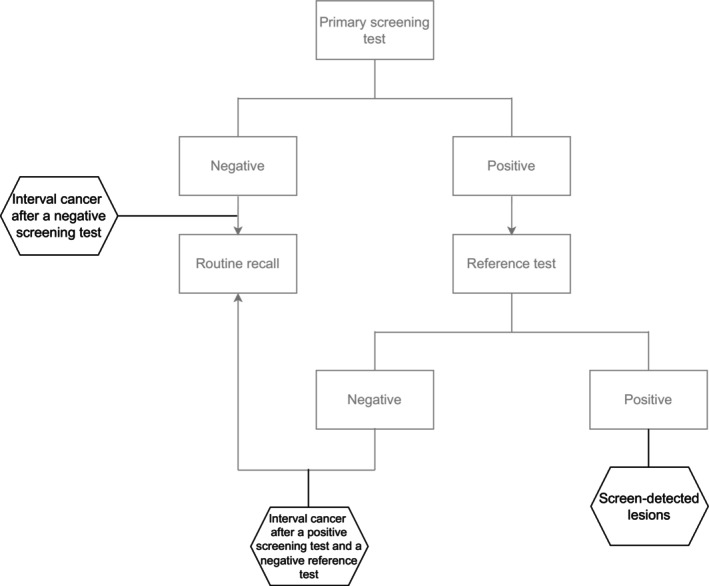
Simplified representation of the typical management of individuals screened for cervical or colorectal cancer, with identification of screen‐detected lesions and lesions missed by screening (interval cancers after negative screening tests and interval cancers after positive tests in whom the reference test was negative). While an individual remains in the target group for a particular type of screening (this is usually capped at a certain age), those with negative screening tests are routinely recalled after the period equal to the screening interval. This has varied between one to 5 years in cytology‐based cervical screening, up to 10 years in HPV‐based cervical screening, around 2 years in FIT‐based colorectal screening, and up to 10 years in colonoscopy‐based colorectal screening. Negative reference tests may be repeated one or more times to form a safety net before the individuals are returned to routine recall.

## Data Availability

All data used for this study are available through electronic references provided in Data S1. Further information is available from the corresponding author upon request.

## APPENDIX A SCREENING FOR CERVICAL AND COLORECTAL CANCERS

Cervical and colorectal screening differ from, for example, breast or lung screening in that a large proportion of lesions are detectable at a preinvasive stage. Not all preinvasive lesions would progress to cancer in the absence of screening.^17^, ^42^, ^129^ Consequently, treatment of non‐progressive lesions is frequent though often undertaken in outpatient clinics without requiring full sedation.^130^

Various countries have successfully implemented cervical screening since the 1960s. Until recently, the main test used for primary screening has been cytology. Cytology requires high‐quality samples taken directly from the cervix that are fixated on a slide and evaluated in laboratories by trained cytoscreeners and cytopathologists. The interpretation of the continuum of cytological abnormalities from slides is, to a degree, subjective. More recently, programmes have started substituting cytology with human papillomavirus (HPV) testing which requires a similar kind of sample but allows for automated evaluation of the presence of viral particles. Here, cytology continues to be used to triage individuals with positive HPV tests. For both cytology and HPV testing, several technologies are commercially available. The accuracy of the available technologies may differ, and each technology may also require specific training of the laboratory personnel.^131^, ^132^, ^133^ Women with positive screening tests are offered a referral to colposcopy; though in some programmes, a referral may only be made after additional tests in early recall. The responsibility for the follow‐up of individuals with positive screening and early recall tests usually does not lie directly with laboratories but with the programme itself or with colposcopy clinics, and the adherence may differ by geographical area.^36^

Population‐based colorectal screening programmes have been established in several European countries in the last 10–15 years.^134^ Screening methods for colorectal cancer include guaiac‐based faecal occult blood (gFOBT) and faecal immunochemical tests (FIT), sigmoidoscopy, and colonoscopy.^135^ The most frequently used colorectal screening test globally is the FIT test,^136^ which detects small traces of blood in the stool from bleeding invasive lesions before these cause other clinical symptoms. Colonoscopy and sigmoidoscopy are more invasive than FIT and gFOBT tests; they examine the entire intestine or a part of it, and are more likely to detect preinvasive polyps and adenomas.^135^, ^136^ Colonoscopy is also used as the reference diagnostic test for individuals with positive FIT or gFOBT test results. Evidence on the efficacy of some of these tests has been documented through randomized controlled trials.^135^, ^137^, ^138^, ^139^

Figure A1 shows the usual clinical pathways for individuals screened for cervical and colorectal cancers.

## APPENDIX B MONITORING GAPS IN DETECTING CASES OF PREVALENT DISEASE USING MEASURE #2 ON ROUTINELY COLLECTED DATA UNDER HYPOTHETICAL SCENARIOS

### B.1 Context

A health care service provides screening for cancer. The screening test employed can detect preinvasive lesions (e.g., cervical intraepithelial neoplasia in cervical screening or advanced adenoma in colorectal screening) and cancer. Preinvasive lesions for this cancer are not normally detected without screening. The screening programme is achieving 100% uptake and uses two screening providers, whose “true” sensitivity for target disease is 60% (provider P1) and 100% (provider P2). Since the individuals with negative screening tests are not referred for reference testing, and the “true” sensitivity of the reference test is also unknown, the service attempts to estimate screening sensitivity using routinely collected data. These data include information on all screening tests, reference tests, and the related diagnoses, which can be linked at the individual level allowing for a correct reconstruction of the sequence of the events.

### B.2 Measure

The service estimates the sensitivity using measure #2 (cf., Table 2), which is an adaptation of the “detection” method where both the numerator and denominator include preinvasive lesions and cancers rather than only invasive cancers.

### B.3 Assumptions

Assume that in the population served by this service, there are N_1_ = 1000 individuals with the prevalent target disease in the screening round for which the sensitivity is being estimated. Assume, furthermore, that among those with negative tests in this round another N_2_ will develop incident (de novo) target lesions by the time of the subsequent screening round. For simplicity, also assume that all individuals with negative screening tests are referred to routine recall, and that none of the target lesions that were missed in the initial round would progress to symptomatic disease during this time (i.e., there are no interval cancers).

### B.4 Hypothetical scenarios

Based on these assumptions, Table B1 provides the estimates of screening sensitivity under the following scenarios:

Scenario 1: Sensitivity of the reference test (SE_ref_) is 100% and reattendance at the next round (reatt) is 100%.

- Scenario 1a: N_2_ = 1000,
- Scenario 1b: N_2_ = 500,
- Scenario 1c: N_2_ = 0.

Scenario 2: as Scenario 1a but reatt = 80%.

Scenario 3: as Scenario 1a but SE_ref_ = 80%.

### B.5 Interpretation of the values in Table B1

Under Scenario 1a, the sensitivity of provider P1 would be estimated as 42% (vs. 60% “true” sensitivity) and that of provider P2 as 50% (vs. 100% “true” sensitivity). With fewer incident lesions in Scenario 1b, the sensitivity for provider P1 would be estimated as 53% (vs. 60% “true” sensitivity) and that of provider P2 as 67% (vs. 100% “true” sensitivity). Had there been no incident lesions after the initial screen as in Scenario 1c, the sensitivity of provider P1 would be estimated as 71% (vs. 60% “true” sensitivity) and that of provider P2 as 100% (vs. 100% “true” sensitivity). When N_2_ = 1000 is held constant, these estimates would differ from those in Scenario 1a when fewer people attend the next screening round (reattendance = 80% in Scenario 2 vs. 100% in Scenario 1a): 47% (vs. 60% “true” sensitivity and 42% in Scenario 1a) for P1 and 56% (vs. 100% “true” sensitivity and 50% in Scenario 1a) for P2. In case the sensitivity of the reference test is lower (80% in Scenario 3 vs. 100% in Scenario 1a), the estimate of screening sensitivity would be 37% (vs. 60% “true” sensitivity and 42% in Scenario 1a) for P1 and 50% (vs. 100% “true” sensitivity and 50% in Scenario 1a) for P2.

**TABLE B1:** Estimates of screening sensitivity under the hypothetical Scenarios 1(a–c), 2, and 3 as defined above.

	“True” sensitivity for target lesions^a^	Screening round for which the sensitivity is estimated		Subsequent screening round		Estimate of sensitivity
		Detected lesions	Missed lesions	Prevalent lesions	Detected lesions	
	A	B = A × N_1_ × SE_ref_	C = N_1_ − B	D = N_2_ + C	E = A × D × SE_ref_ × reatt	F = B/(B + E)
Scenario 1a						
Provider P1	60%	600	400	1400	840	42%
Provider P2	100%	1000	0	1000	1000	50%
Scenario 1b						
Provider P1	60%	600	400	900	540	53%
Provider P2	100%	1000	0	500	500	67%
Scenario 1c						
Provider P1	60%	600	400	400	240	71%
Provider P2	100%	1000	0	0	0	100%
Scenario 2						
Provider P1	60%	600	400	1400	672	47%
Provider P2	100%	1000	0	1000	800	56%
Scenario 3						
Provider P1	60%	480	400	1400	672	37%
Provider P2	100%	800	0	1000	800	50%

Abbreviations: N_1_, number of individuals with prevalent lesions (equals 1000 in all scenarios); N_2_, number of individuals with de novo incident lesions (1000 under scenarios 1a, 2, and 3; 500 under Scenario 1b, and 0 under Scenario 1c); reatt, reattendance at the subsequent screening round; SE_ref_, sensitivity of reference testing.

^a^ The sensitivity that the screening service tries to estimate using routinely collected data.

## APPENDIX C MONITORING GAPS IN DETECTING CASES OF PREVALENT DISEASE USING MEASURE #3 ON ROUTINELY COLLECTED DATA UNDER HYPOTHETICAL SCENARIOS

### C.1 Context

As in Appendix B.

### C.2 Measure

The service estimates the sensitivity using measure #3 (cf., Table 2). This is an adaptation of the “detection” method in which the definition of the target disease is changed so that true‐positive tests include detection of preinvasive lesions or worse but continue to record false‐negative tests as interval cancers.

### C.3 Assumptions

Assume that in the population served by this service, there are N_1_ = 1000 individuals with the prevalent target disease (preinvasive lesions or cancer) in the screening round for which the sensitivity is being estimated. Assume, furthermore, that a proportion of preinvasive lesions that were missed at the first round progresses to symptomatic cancer before the next screening round. Although this proportion cannot be observed directly, the absolute number of resulting cancers can be observed as long as the affected individuals present for diagnosis promptly, the diagnostic services are highly accurate, and the lookout period is adequate. Assume, finally, that among those with negative tests in this round N_2_ = 10 will develop incident (de novo) cancer before the next screen which, in the routinely collected data, are indistinguishable from the true interval cancers; and that these symptomatic cancers are diagnosed once the individual presents with symptoms.

### C.4 Hypothetical scenarios

Based on these assumptions, Table C1 provides the estimates of screening sensitivity under the following scenarios:

Scenario 1: Sensitivity of the reference test (SE_ref_) = 100%.

- Scenario 1a: Proportion of preinvasive lesions that progress to cancer (prog) = 0%,
- Scenario 1b: prog = 5%,
- Scenario 1c: prog = 50%,
- Scenario 1d: prog = 100%.

Scenario 2: As Scenario 1b but SE_ref_ = 80%.

### C.5 Interpretation of the values in Table C1

Under Scenario 1a, the sensitivity of provider P1 would be estimated as 98% (vs. 60% “true” sensitivity) and that of provider P2 as 99% (vs. 100% “true” sensitivity). There is little variation in these estimates except in Scenarios 1c and 1d for P1, which assume lesion progression rates that are unrealistic for cervical or colorectal screening.

**TABLE C1:** Estimates of screening sensitivity for hypothetical Scenarios 1(a–c) and 2, as defined above.

	“True” sensitivity for target lesions^a^	Screening round for which the sensitivity is estimated		Missed lesions that will progress to cancer before the next screening round	Estimate of sensitivity
		Detected lesions	Missed lesions		
	A	B = N_1_ × A × SE_ref_	C = N_1_ − B	D = C × prog	E = B/(B + D + N_2_)
Scenario 1a					
Provider P1	60%	600	400	0	98%
Provider P2	100%	1000	0	0	99%
Scenario 1b					
Provider P1	60%	600	400	20	95%
Provider P2	100%	1000	0	0	99%
Scenario 1c					
Provider P1	60%	600	400	200	74%
Provider P2	100%	1000	0	0	99%
Scenario 1d					
Provider P1	60%	600	400	400	59%
Provider P2	100%	1000	0	0	99%
Scenario 2					
Provider P1	60%	480	400	20	94%
Provider P2	100%	800	0	0	99%

Abbreviations: N_1_, number of individuals with prevalent lesions (equals 1000 in all scenarios); N_2_, number of individuals with de novo interval cancers (equals 10 in all scenarios); prog, the proportion of individuals with missed lesions in the first round who progress to cancer before the next screening round; SE_ref_, sensitivity of reference testing.

^a^ The sensitivity that the screening service tries to estimate using routinely collected data.

## APPENDIX D MONITORING GAPS IN DETECTING CASES OF PREVALENT DISEASE USING MEASURE #10 ON ROUTINELY COLLECTED DATA UNDER HYPOTHETICAL SCENARIOS

### D.1 Context

As in Appendix B.

### D.2 Measure

The service identifies issues with the detection of cases of prevalent disease among its screening providers using measure #10 (cf., Table 2). This measure includes interval cancers diagnosed among the individuals with negative screening tests in the numerator, and the number of individuals with negative screening tests in the denominator.

### D.3 Assumptions

Assume that *N* = 100,000 individuals are screened under each scenario. In an average‐risk population, 0.1% (i.e., 100 individuals) have a prevalent target cancer, all of which would progress to a symptomatic stage and represent true interval cancers if not diagnosed at screening, and 0.01% (i.e., 10 individuals) develop a de novo symptomatic cancer during the screening interval. In a low‐risk population, these values are 90% lower, whereas in a high‐risk population, these values are twice as high. Assume also that the proportion of the screened population who have a positive test varies with test sensitivity; that the positive predictive value (PPV) for cancer among individuals with a positive screening test is 1% in all scenarios, and that for simplicity this is constant; and that the sensitivity of the reference test is 100%.

### D.4 Hypothetical scenarios

Based on these assumptions, Table D1 provides estimates using measure #10 under the following scenarios:

Scenario 1: Average‐risk population (prevalence (prev) = 0.1%, incidence (inc) = 0.01%).

Scenario 2: Low‐risk population (prev = 0.01%, inc = 0.001%).

Scenario 3: High‐risk population (prev = 0.2%, inc = 0.02%).

### D.5 Interpretation of the values in Table D1

As expected, the observed interval cancer rate in individuals with negative screens increases when the “true” test sensitivity is lower. It also increases when the background risk of cancer (i.e., the incidence rate that would be observed had there been no screening or another type of prevention such as HPV vaccination in the case of cervical cancer) is higher even when the “true” test sensitivity remains the same.

**TABLE D1:** The observed interval cancer rates in hypothetical Scenarios 1–3, as defined above.

	“True” sensitivity for target lesions^a^	Prevalent cancers	Negative screening tests	Screen‐detected cancers	True interval cancers	De novo target cancers	Interval cancer rate per 100,000 negative screens
	A	B = N × prev	C = N × (1 − prev × A/PPV)	D = A × B	E = B − D	F = N × inc	G = (E + F) × 100,000/C
Scenario 1							
Provider P1	60%	100	94,000	60	40	10	53.2
Provider P2	100%	100	90,000	100	0	10	11.1
Scenario 2							
Provider P1	60%	10	99,400	6	4	1	5.0
Provider P2	100%	10	99,000	10	0	1	1.0
Scenario 3							
Provider P1	60%	200	88,000	120	80	20	113.6
Provider P2	100%	200	80,000	200	0	20	25.0

Abbreviations: N, number of screened individuals (equals 100,000 in all scenarios); inc, proportion of individuals with de novo cancer (equals 10% of the prevalence in all scenarios); PPV, positive predictive value for cancer among individuals with a positive screening test (equals 1% in all scenarios); prev, the proportion of the screened population with a prevalent cancer.

^a^ With measure #10, there is no attempt to estimate the “true” sensitivity directly. The lower the “true” sensitivity, however, the higher the interval cancer rate will be. Hence, high values of the interval cancer rate are interpreted as suggestive of issues with test sensitivity (e.g., when comparing the values for the same provider over time, or when comparing between providers).

## APPENDIX E MONITORING GAPS IN DETECTING CASES OF PREVALENT DISEASE USING MEASURE #16 ON ROUTINELY COLLECTED DATA UNDER HYPOTHETICAL SCENARIOS

### E.1 Context

As in Appendix B.

### E.2 Measure

The service identifies issues with the detection of cases of prevalent disease among its screening providers using measure #16 (cf., Table 2). This measure monitors the number of detected target lesions (usually defined as a combination of preinvasive diagnoses and cancers, but could also include only cancers) in the numerator among all screened individuals in the denominator.

### E.3 Assumptions

Assume that providers P1 and P2 offer screening to areas with 100,000 individuals with three different levels of prevalence of the target disease (low: 0.5%, average: 1%, and high: 2%); and that the sensitivity of the reference test is 100%.

### E.4 Hypothetical scenarios

Based on these assumptions, Table E1 provides estimates for measure #16 under the following scenarios:

Scenario 1: Low‐risk population (prevalence (prev) = 0.5%).

Scenario 2: Average‐risk population (prev = 1.0%).

Scenario 3: High‐risk population (prev = 2.0%).

### E.5 Interpretation of the values in Table E1

The observed detection rate increases when the “true” test sensitivity is higher. It also increases when the background risk of target lesions (i.e., the incidence rate that would be observed had there been no screening or another type of prevention such as HPV vaccination in the case of cervical cancer) is higher even when the “true” test sensitivity remains the same.

**TABLE E1:** The observed detection rates in hypothetical Scenarios 1–3, as defined above.

	“True” sensitivity for target lesions^a^	Detected target lesions	Detection rate of the target lesions (per 1000 screened)
	A	B = A × N × prev	C = B × 1000/N
Scenario 1			
Provider P1	60%	300	3.0
Provider P2	100%	500	5.0
Scenario 2			
Provider P1	60%	600	6.0
Provider P2	100%	1000	10.0
Scenario 3			
Provider P1	60%	1200	12.0
Provider P2	100%	2000	20.0

Abbreviations: N, number of screened individuals (equals 100,000 in all scenarios); prev, the proportion of the screened population with a prevalent lesion.

^a^ With this measure (#16), there is no attempt to estimate the “true” sensitivity directly. The lower the “true” sensitivity, however, the lower the detection rate will be. Hence, low values of the detection rate are interpreted as suggestive of issues with test sensitivity (e.g., when comparing the values for the same provider over time, or when comparing between providers).
