# Supplementary material for: Indicators to identify cancer screening providers with suboptimal case detection: A scoping review
Source: Int J Cancer. 2025 Nov 29;158(9):2339–58. doi: 10.1002/ijc.70264 (PMC12963708; doi:10.1002/ijc.70264)
Supplement: Supplementary file 1 — Data S1. Supporting Information. [file IJC-158-2339-s001.pdf]

INDICATORS TO IDENTIFY CANCER SCREENING PROVIDERS WITH SUBOPTIMAL CASE DETECTION: A SCOPING REVIEW

SUPPLEMENTARY MATERIAL

Jiayao Lei, Milena Falcaro, Adam R Brentnall,
James F O'Mahony, Sisse Helle Njor, Matejka Rebolj

Table of contents:

References for Cervical Screening Programmes
References for Colorectal Screening Programmes

The list of countries representing Europe included in our scoping review followed that used by Bruni and colleagues and included 43 countries.[a] Note, only a proportion of the 43 countries have implemented organised screening programmes.

The lists below include documents that were consulted for each of these countries, for cervical and colorectal cancer separately. They include:

- reports that provide information stating or suggesting whether the country has an organised screening programme.
- annual statistical reports in the form of published documents or summary statistics from the programme websites, and other documents which provided data for Table 2;
- statistical reports that do not include indicators of screening sensitivity: if this was the only report of its kind that our search could identify for a given country, it was taken as suggesting that a country does not systematically monitor the ability of the providers to detect prevalent disease;

National documents were prioritised; if none could be found, we consulted country-specific documents that have been made available by supranational institutions such as IARC.

[a] Bruni L, Serrano B, Roura E, Alemany L, Cowan M, Herrero R, Poljak M, Murillo R, Broutet N, Riley LM, de Sanjose S. Cervical cancer screening programmes and age-specific coverage estimates for 202 countries and territories worldwide: a review and synthetic analysis. Lancet Glob Health 2022;10: e1115-e27.

REFERENCES FOR CERVICAL SCREENING PROGRAMMES

Albania

- Albania national cervical cancer program evaluation report of the first year 2020. https://www.ishp.gov.al/wp-content/uploads/2021/06/2report-evaluation-screening-program-final-2020.pdf (accessed 23 August 2022)

Andorra

- Andorra National Health Survey 2011. https://ghdx.healthdata.org/record/andorra-national-health-survey-2011 (accessed 23 August 2022)

Austria

- Tsibulak I, Reiser E, Bogner G, et al. Decrease in gynecological cancer diagnoses during the COVID-19 pandemic: an Austrian perspective. Int J Gynecol Cancer. 2020;30(11):1667-1671. doi:10.1136/ijgc-2020-001975 https://ijgc.bmj.com/content/ijgc/30/11/1667.full.pdf

Belarus

- Bruni L, Albero G, SerranoB et al. ICO/IARC Information Centre on HPV and Cancer (HPV Information Centre). Human Papillomavirus and Related Diseases Report in Belarus. Summary Report 10 March 2023. https://hpvcentre.net/statistics/reports/BLR.pdf (accessed 23 April 2023)

Belgium

- Flanders, Annual report 2014 https://baarmoederhalskanker.bevolkingsonderzoek.be/sites/default/files/2022-03/Jaarrapport%202014.pdf (accessed 14 March 2023)
- Flanders, Annual report 2015 https://baarmoederhalskanker.bevolkingsonderzoek.be/sites/default/files/2022-03/Jaarrapport%202015.pdf (accessed 14 March 2023)
- Flanders, Annual report 2016 https://baarmoederhalskanker.bevolkingsonderzoek.be/sites/default/files/2022-03/Jaarrapport2016.pdf (accessed 14 March 2023)
- Flanders, Annual report 2017 https://baarmoederhalskanker.bevolkingsonderzoek.be/sites/default/files/2022-03/Jaarrapport2017.pdf (accessed 14 March 2023)
- Flanders, Annual report 2018 https://baarmoederhalskanker.bevolkingsonderzoek.be/sites/default/files/2022-03/Jaarrapport%202018_CvKO_SKR_0.pdf (accessed 14 March 2023)
- Flanders, Annual report 2019 https://baarmoederhalskanker.bevolkingsonderzoek.be/sites/default/files/2022-03/Jaarrapport2019_0.pdf (accessed 14 March 2023)
- Flanders, Annual report 2020 https://baarmoederhalskanker.bevolkingsonderzoek.be/sites/default/files/2022-03/Jaarrapport%202020_0_0.pdf (accessed 14 March 2023)
- Flanders, Annual report 2021 https://baarmoederhalskanker.bevolkingsonderzoek.be/sites/default/files/2022-03/CVK21-0027%20Berekeningswijze%20BHK-v1%5B1%5D%5B1%5D.pdf (accessed 14 March 2023)
- Flanders, Annual report 2022 https://baarmoderhalskanker.bevolkingsonderzoek.be/sites/default/files/2022-12/Jaarrapport%202022.pdf (accessed 14 March 2023)

- Cervical Cancer Population Screening Definitions and calculation methods 2020. https://baarmoederhalskanker.bevolkingsonderzoek.be/sites/default/files/2022-04/Berekeningswijze_BHK_JR2019.pdf (accessed 14 March 2023)
- Cervical Cancer Population Screening Definitions and calculation methods 2021. https://baarmoederhalskanker.bevolkingsonderzoek.be/sites/default/files/2022-03/CVK21-0027%20Berekeningswijze%20BHK-v1%5B1%5D%5B1%5D.pdf (accessed 14 March 2023)
- Cervical Cancer Population Screening Definitions and calculation methods 2022. https://baarmoederhalskanker.bevolkingsonderzoek.be/sites/default/files/2022-12/Berekeningswijze%20Jaarrapport%202022%20Baarmoederhalskanker.pdf (accessed 14 March 2023)

Bosnia and Herzegovina

- Bosnia and Herzegovina cervical cancer profile. https://www.iccp-portal.org/system/files/plans/cervical-cancer-bih-2021-country-profile-en.pdf (accessed 20 April 2022)

Bulgaria

- Karcheva M, Yordanov A, Kostadinov S. An overview of cervical cancer epidemiology and prevention in Bulgaria. Germs. 2020 Dec 28;10(4):322-327. doi: 10.18683/germs.2020.1224. PMID: 33489947; PMCID: PMC7811861.
- Bulgaria Human Papillomavirus and Related Cancers, FactSheet2023. https://hpvcentre.net/statistics/reports/BGR_FS.pdf (accessed 20 April 2023)

Croatia

- National cancer control plan 2020 – 2030. https://www.iccp-portal.org/system/files/plans/NPPR_ENG_final.pdf (accessed 23 August 2022)
- Vrdoljak-Mozetic D, Ostojić DV, Stemberger-Papić S, et al. Cervical cancer screening programme in Primorsko-Goranska County, Croatia--the results of the pilot study. *Coll Antropol*. 2010;34(1):225-232.

Cyprus

- Cervical cancer Cyprus 2021 country profile. https://www.who.int/publications/m/item/cervical-cancer-cyp-country-profile-2021 (accessed 23 August 2022)

Czechia

- Selected publications referring to cervical cancer screening programme in the Czech Republic. https://www.cervix.cz/en/professionals/publications/ (accessed 20 April 2022)
- Screening process. https://www.cervix.cz/en/professionals/screening-process/ (accessed 20 April 2022)
- Májek, Ondřeja,c; Dušková, Jaroslavad; Dvořák, Vladimírb et al. Performance indicators in a newly established organized cervical screening programme: registry-based analysis in the Czech Republic. European Journal of Cancer Prevention 26(3):p 232-239, May 2017. | DOI: 10.1097/CEJ.0000000000000236 https://journals.lww.com/eurjcancerprev/pages/articleviewer.aspx?year=2017&issue=05000&article=00008&type=Fulltext

Denmark

- Dansk Kvalitetsdatabase for Livmoderhalskræftscreening Årsrapport 2014 https://danskcytologiforening.dk/dkls/2014/DKLS%202014.pdf (accessed 20 April 2022)

- Dansk Kvalitetsdatabase for Livmoderhalskræftscreening Årsrapport 2015 https://danskcytologiforening.dk/dkls/2015/DKLS%202015.pdf (accessed 20 April 2022)
- Dansk Kvalitetsdatabase for Livmoderhalskræftscreening Årsrapport 2016 https://danskcytologiforening.dk/dkls/2016/DKLS%202016.pdf (accessed 20 April 2022)
- Dansk Kvalitetsdatabase for Livmoderhalskræftscreening Årsrapport 2017 https://danskcytologiforening.dk/dkls/2017/DKLS%202017.pdf (accessed 20 April 2022)
- Dansk Kvalitetsdatabase for Livmoderhalskræftscreening Årsrapport 2018 https://danskcytologiforening.dk/dkls/2018/DKLS_aarsrapport_2018.pdf (accessed 20 April 2022)
- Dansk Kvalitetsdatabase for Livmoderhalskræftscreening Årsrapport 2019 https://danskcytologiforening.dk/dkls/2019/dkls_aarsrapport_2019.pdf (accessed 20 April 2022)
- Dansk Kvalitetsdatabase for Livmoderhalskræftscreening Årsrapport 2020 https://danskcytologiforening.dk/dkls/2020/dkls_aarsrapport_2020_offentlig_version.pdf (accessed 20 April 2022)
- Dansk Kvalitetsdatabase for Livmoderhalskræftscreening Årsrapport 2022 https://www.sundhed.dk/content/cms/82/4682_dkls-aarsrapport-2022.pdf (accessed 20 April 2022)

Estonia

- CANCER SCREENING FACT SHEET (3 PAGES) ESTONIA – CERVIX – 2017 https://nordscreen.org/wp-content/uploads/2017/07/cervix-fact-sheet-estonia-2017.pdf (accessed 24 August 2022)
- Cervical cancer screening. https://www.itk.ee/en/cervical-cancer-screening (accessed 24 August 2022)
- Screening statistics. https://statistika.tai.ee/pxweb/et/Andmebaas/Andmebaas__02Haigestumus__07Soeluuringud/VSR17.px/ (accessed 24 August 2022)

Finland

- Cervical cancer screening programme in Finland. Annual review 2021. https://syoparekisteri.fi/assets/files/2021/09/The-Cervical-Cancer-Screening-Programme_Annual-review_2021.pdf (accessed 24 August 2022)

France

- Cervical cancer screening. https://www.e-cancer.fr/Professionnels-de-sante/Depistage-et-detection-precoce/Depistage-du-cancer-du-col-de-l-uterus (accessed 16 March 2023)

Germany

- Cervical cancer screening. Concept of a tiered evaluation (30 Nov 2017). https://www.g-ba.de/downloads/40-268-6040/2018-11-22_oKFE-RL_Zervixkarzinom_IQTIG-Konzept.pdf (last accessed 24 August 2022)
- The Joint Federal Committee for Organized Early Cancer Detection Programs. https://www.g-ba.de/downloads/62-492-3039/oKFE-RL-2022-11-17-iK-2023-01-26.pdf (last accessed 24 August 2022)

Greece

- Eurostat – Health care activities. Screening. Definitions. https://ec.europa.eu/eurostat/cache/metadata/Annexes/hlth_ps_scre_esms_an1.pdf (last accessed 24 August 2022)

Hungary

- VAJDA RÉKA, ÁRVÁNÉ EGRI CSILLA, KOVÁCS ATTILA et al. Quality indicators and performance indicators of the nurse cervical screening pilot program. 2017. https://huon.hu/2017/61/4/0361/0361a.pdf (last accessed 20 April 2022)

Iceland

- Cancer screening. https://www.krabb.is/skimun/krabbameinsleit/ (last accessed 20 April 2022)

Ireland

- Interval Cancer Report CervicalCheck 2020. https://assets.hse.ie/media/documents/interval-cancer-report-cervicalcheck.pdf (accessed 24 August 2022)
- Implementation of recommendations arising from the Interval Cancer Expert Reference Group Reports. Annual Report 2020/2021 Year1. https://www.screeningservice.ie/publications/NSS-Interval-Cancer-Project-Year1-Nov-2021.pdf (accessed 24 August 2022)
- CervicalCheck Tribunal annual report 2020. http://hdl.handle.net/10147/630899. (accessed 24 August 2022)
- CervicalCheck Programme Report 2016-2017. https://www.screeningservice.ie/publications/CervicalCheck-Programme-Report-2016-2017.pdf (accessed 24 August 2022)
- CervicalCheck Programme Report 2014-2015. https://www.cervicalcheck.ie/_fileupload/ProgrammeReports/CS-PR-PM-20%20CervicalCheck%20Programme%20Report%202014-2015.pdf (accessed 24 August 2022)
- CervicalCheck Programme Report 2012-2013. https://assets.hse.ie/media/documents/CervicalCheck_Programme_Report_2012_-_2013.pdf (accessed 24 August 2022)
- CervicalCheck Programme Report 2011-2012. https://assets.hse.ie/media/documents/CervicalCheck_Programme_Report_2011_-_2012.pdf (accessed 24 August 2022)
- Cervical check programme report 1 September 2010 – 31 August 2011 http://hdl.handle.net/10147/248833 (accessed 24 August 2022)
- CervicalCheck programme report 2008-2009. http://hdl.handle.net/10147/145908 (accessed 24 August 2022)
- Programme report 2007 Irish Cervical Screening Programme. http://hdl.handle.net/10147/76803 (accessed 24 August 2022)
- Irish Cervical Screening Programme annual report 2006. https://www.cervicalcheck.ie/_fileupload/Publications/ICSP_Annual_Report_2006_23128496.pdf (accessed 24 August 2022)
- Irish Cervical Screening Programme annual report 2005. https://www.lenus.ie/handle/10147/45968 (accessed 24 August 2022)
- Standards for Quality Assurance in Cervical Screening Standards for Quality Assurance in Colposcopy. https://www.cervicalcheck.ie/_fileupload/QualityAssurance/Quality%20Assurance%20in%20Colposcopy%20Dec%202020%20(P41).pdf (accessed 24 August 2022)
- Standards for Quality Assurance in Cervical Screening. Quality assurance in programme operation. https://www.cervicalcheck.ie/_fileupload/QualityAssurance/Quality%20assurance%20in%20programme%20operation.pdf (accessed 24 August 2022)

Italy

- Cervical screening. Activity data from 2017 to 2021. https://www.osservatori-onazionalescreening.it/content/lo-screening-cervicale (accessed 24 August 2022)
- National screening observatory Report on 2019. https://www.osservatorionazionalescreening.it/sites/default/files/allegati/Rapportosul2019.pdf:
- Ronco G, Giubilato P, Carozzi F, et al. Extension of organized cervical cancer screening programmes in Italy and their process indicators, 2011-2012 activity. *Epidemiol Prev*. 2015;39(3 Suppl 1):61-76. https://pubmed.ncbi.nlm.nih.gov/26405778/
- Ronco G, Giubilato P, Naldoni C, et al. Estensione dei programmi organizzati di screening del cancro cervicale in Italia e loro indicatori di processo, attività 2010 [Extension of organised cervical cancer screening programmes in Italy and their process indicators, 2010 activity]. *Epidemiol Prev*. 2012;36(6 Suppl 1):39-54. https://pubmed.ncbi.nlm.nih.gov/23293270/
- Ronco G, Giubilato P, Naldoni C, et al. Extension of organised cervical cancer screening programmes in Italy and their process indicators, 2009 activity. Epidemiol Prev. 2011;35(5-6 Suppl 5):39-54. https://pubmed.ncbi.nlm.nih.gov/22166349/
- Ronco G, Giubilato P, Naldoni C, et al. Extension of organised cervical cancer screening programmes in Italy and their process indicators: 2008 activity. Epidemiol Prev. 2010;34(5-6 Suppl 4):35-51. https://pubmed.ncbi.nlm.nih.gov/21220836/
- Ronco G, Giubilato P, Naldoni C, et al. Extension of organised cervical cancer screening programmes in Italy and their process indicators: 2007 activity. *Epidemiol Prev*. 2009;33(3 Suppl 2):41-56. https://pubmed.ncbi.nlm.nih.gov/19776486/
- Ronco G, Giubilato P, Naldoni C, et al. Extension of organised cervical cancer screening programmes in Italy and their process indicators. *Epidemiol Prev*. 2008;32(2 Suppl 1):37-54. https://pubmed.ncbi.nlm.nih.gov/18770994/
- Ronco G, Giubilato P, Naldoni C, et al. Extension of organised cervical cancer screening programmes in Italy and their process indicators. *Epidemiol Prev*. 2007;31(2-3 Suppl 2):33-47. https://pubmed.ncbi.nlm.nih.gov/17824361/
- Ronco G, Giubilato P, Naldoni C, et al. Activity level and process indicators of organised programmes for cervical cancer screening in Italy. *Epidemiol Prev*. 2006;30(1 Suppl 3):27-40. https://pubmed.ncbi.nlm.nih.gov/16937844/

Latvia

- Kojalo U, Tisler A, Parna K, Kivite-Urtane A, Zodzika J, Stankunas M, Baltzer N, Nygard M, Uuskula A. An overview of cervical cancer epidemiology and prevention in the Baltic States. BMC Public Health. 2023 Dec;23(1):1-9. https://assets.researchsquare.com/files/rs-1831455/v1/17caa6c5-8241-4117-8820-217282015d78.pdf?c=1659376599

Lithuania

- Kojalo U, Tisler A, Parna K, Kivite-Urtane A, Zodzika J, Stankunas M, Baltzer N, Nygard M, Uuskula A. An overview of cervical cancer epidemiology and prevention in the Baltic States. BMC Public Health. 2023 Dec;23(1):1-9. https://assets.researchsquare.com/files/rs-1831455/v1/17caa6c5-8241-4117-8820-217282015d78.pdf?c=1659376599

Luxembourg

- Gynaecological cytology service – LNS. https://lns.lu/en/departement/department-of-anatomic-and-molecular-pathology/gynaecological-cytology-service/ (accessed 25 August 2022)

Malta

- Scope of cervical screening programme. https://healthservices.gov.mt/en/phc/nbs/Pages/Screening-Programmes/Cervical-Screening.aspx (accessed 25 August 2022)

Monaco

- The Monaco Health Screening Centre – An exemplary preventative health initiative. https://en.gouv.mc/Policy-Practice/Social-Affairs-and-Health/An-exemplary-Public-Health-system/Monaco-Health-Screening-Centre (accessed 25 August 2022)
- Screening for cervical cancer. https://monservicepublic.gouv.mc/en/themes/social-health-and-families/public-health/prevention-and-screening/screening-for-cervical-cancer (accessed 25 August 2022)

Montenegro

- National programme for early detection of cervical cancer. https://www.skriningsrbija.rs/files/File/Nacionalni_program_ranog_otkrivanja_karcinoma_grlica_materice.pdf (accessed 25 August 2022)
- Lukač A, Šulović N, Smiljić S, Ilić AN, Šašić M. HPV Typing as a Screening Program for Prevention and Early Detection of Cervical Cancer in Different Montenegro Regions. Acta Clinica Croatica. 2020 Jun;59(2):294.
- National Cervical Cancer Screening begins. https://www.gov.me/cyr/clanak/181714--pocinje-nacionalni-skrining-karcinoma-grlica-materice (accessed 25 August 2022)

The Netherlands

- Monitoring and evaluation. https://www.rivm.nl/bevolkingsonderzoek-baarmoederhalskanker/professionals/monitoring-en-evaluatie (accessed 25 August 2022)
- National evaluation of the population screening for cervical cancer 2017-2020. https://pure.eur.nl/ws/portalfiles/portal/61846660/LEBA2020_NL_def.pdf (accessed 25 August 2022)
- Indicators renewed BVO BMHK. https://www.rivm.nl/sites/default/files/2018-11/Indicatoren%20totaal%20v0.6.pdf (accessed 25 August 2022)
- Cervical cancer population screening monitor 2021. https://www.rivm.nl/sites/default/files/2022-10/Monitor%20bevolkingsonderzoek%20baarmoederhalskanker%202021.pdf (accessed 25 August 2022)
- Cervical cancer population screening monitor 2020. https://www.rivm.nl/sites/default/files/2021-09/IKNL_monitor-BMHK-160921.pdf (accessed 25 August 2022)
- Cervical cancer population screening monitor 2019. https://www.rivm.nl/documenten/monitor-bevolkingsonderzoek-baarmoederhalskanker-2019 (accessed 25 August 2022)
- Cervical cancer population screening monitor 2018. https://www.rivm.nl/documenten/monitor-bevolkingsonderzoek-baarmoederhalskanker-2018 (accessed 25 August 2022)
- Cervical cancer population screening monitor 2017. https://www.rivm.nl/documenten/landelijke-evaluatie-van-bevolkingsonderzoek-baarmoederhalskanker-leba-tm-2017 (accessed 25 August 2022)

North Macedonia

- Cervical cancer screening in North Macedonia. https://ecancer.org/en/video/7954-cervical-cancer-screening-in-north-macedonia (accessed 25 August 2022)

Norway

- Cervical screening programme. https://www.kreftregisteret.no/screening/livmorhalsprogrammet/Om-programmet/ (accessed 25 August 2022)
- Annual report for cervical screening programme. https://www.kreftregisteret.no/screening/livmorhalsprogrammet/Helsepersonell/ArsrapportLP/ (accessed 25 August 2022)
- Annual report 2021. Screening activity and results from the Cervical Program. https://www.kreftregisteret.no/globalassets/livmorhalsprogrammet/rapporter/arsrapport-lp/arsrapportlp_2021_final2.pdf (accessed 25 August 2022)
- Annual report 2020. https://www.kreftregisteret.no/globalassets/livmorhalsprogrammet/rapporter/arsrapport-lp/arsrapportlp_2020_versjon2.pdf (accessed 25 August 2022)
- Annual report 2019. https://www.kreftregisteret.no/globalassets/livmorhalsprogrammet/rapporter/arsrapport-lp/arsrapport-livmorhalsprogrammet-2019v2_sept2021.pdf (accessed 25 August 2022)
- Annual report 2017-2018. https://www.kreftregis. teret.no/globalassets/livmorhalsprogrammet/rapporter/arsrapport-lp/livmorhals-2017-18.pdf (accessed 25 August 2022)
- Annual report 2016. https://www.kreftregisteret.no/globalassets/livmorhalsprogrammet/rapporter/arsrapport-lp/livmorhals_2016-1-4_rev051118.pdf (accessed 25 August 2022)
- Annual report 2015. https://www.kreftregisteret.no/globalassets/livmorhalsprogrammet/rapporter/arsrapport-lp/aarsrapport_livmorhalsprogrammet-2015.pdf (accessed 25 August 2022)
- Annual report 2013-2014. https://www.kreftregisteret.no/globalassets/livmorhalsprogrammet/rapporter/arsrapport-lp/livmorhals_2015.pdf (accessed 25 August 2022)
- Annual report 2012. https://www.kreftregisteret.no/globalassets/livmorhalsprogrammet/rapporter/arsrapport-lp/aarsrapport_livmorhals_2012.pdf (accessed 25 August 2022)
- Annual report 2008. https://www.kreftregisteret.no/globalassets/livmorhalsprogrammet/rapporter/arsrapport-lp/arsrapport_cervix2008.pdf (accessed 25 August 2022)

Poland

- Maryla Turkot, Dagmara Mokwa, Paulina Wieszczy et al. External audit of providers of the Cervical Cancer Prevention Programme in Poland in 2016/2017. NOWOTWORY J Oncol 2018; 68, 2: 65–78. https://journals.viamedica.pl/nowotwory_journal_of_oncology/article/view/NJO.2018.0011/45179
- Nowakowski A, Arbyn M, Turkot MH, et al. A roadmap for a comprehensive control of cervical cancer in Poland: integration of available solutions into current practice in primary and secondary prevention. *Eur J Cancer Prev*. 2020;29(2):157-164. doi:10.1097/CEJ.0000000000000528

Portugal

- M Correia and others, Algarve Cervical Cancer Screening Programme, *European Journal of Public Health*, Volume 29, Issue Supplement_4, November 2019, ckz186.687, https://doi.org/10.1093/eurpub/ckz186.687

Republic of Moldova

- Cervical screening in Moldova. https://thebiomedicalscientist.net/science/cervical-screening-moldova (accessed 25 August 2022)

Romania

- Todor RD, Bratucu G, Moga MA, Candrea AN, Marceanu LG, Anastasiu CV. Challenges in the Prevention of Cervical Cancer in Romania. *Int J Environ Res Public Health*. 2021;18(4):1721. Published 2021 Feb 10. doi:10.3390/ijerph18041721

Russian Federation

- Andrej M Grjibovski and others, Incidence, mortality and determinants of survival from cervical cancer in Northwest Russia: a registry-based cohort study, *International Health*, Volume 10, Issue 2, March 2018, Pages 92–99, https://doi.org/10.1093/inthealth/ihx068

San Marino

- Prevention screening. https://www.iss.sm/on-line/home/menudestra/screening-prevenzione.html (accessed 26 August 2022)

Serbia

- Results of the six-year cycle of the implementation of an organized cervical cancer-screening program. http://www.dzpozarevac.rs/demo/Skrining2019/Rezultati%20prva%20dva%20ciklusa.docx (accessed 25 August 2022)
- Screening – Serbiancancer. https://serbiancancer.org/skrining/ (accessed 25 August 2022)

Slovakia

- Cancer Screening Programs in Slovakia. Report for 2020. https://www.noisk.sk/files/2021/2021-08-26-noi-rocenka-skriningy-2020-en.pdf (accessed 26 August 2022)
- Started cervical cancer screening. https://www.health.gov.sk/Clanok?odstartoval-skrining-karcinomu-krcka-maternice-2021 (accessed 26 August 2022)

Slovenia

- Annual report 2003-2020. https://zora.onko-i.si/publikacije/letna-porocila (accessed 26 August 2022)
- Annual report on the results of 2020. https://zora.onko-i.si/fileadmin/user_upload/publikacije/izobrazevanja/2021_11ZD_zbornik/1_UrskaI_Porocilo_o_rezultatih_TFkoncna_FINAL.pdf (accessed 26 August 2022)
- Work Report 2020. https://zora.onko-i.si/fileadmin/user_upload/publikacije/izobrazevanja/2021_11ZD_zbornik/2_UrskaI_Opravljeno_delo_TFkoncna_FINAL.pdf (accessed 26 August 2022)
- Annual report 2019 with additional analysis of the results of the ZORA programme during the COVID-19 pandemic in 2020. https://zora.onko-i.si/fileadmin/user_upload/publikacije/izobrazevanja/2020_10ZD_zbornik/1._objava/1-_UrskaI_ZD_Rezultati_progra_ZORA_2019_in_v_casu_pandmije_OCVID-19_2020_koncna_1.pdf (accessed 26 August 2022)
- Annual report 2018. https://zora.onko-i.si/fileadmin/user_upload/publikacije/izobrazevanja/2019_09ZD_zbornik/1._Urska_Ivanus_Porocilo_o_rezultatih_ZORA_-_Copy.pdf (accessed 26 August 2022)
- Annual report 2017. https://zora.onko-i.si/fileadmin/user_upload/publikacije/izobrazevanja/2018_08ZD_zbornik/8ZD_ZBORNIK_015-027.pdf (accessed 26 August 2022)
- Annual report 2015-2016. https://zora.onko-i.si/fileadmin/user_upload/publikacije/izobrazevanja/2017_07ZD_zbornik/7ZD_zbornik_007-011.pdf (accessed 26 August 2022)

- Annual report 2014. https://zora.onko-i.si/fileadmin/user_upload/publikacije/izobrazevanja/2015_06ZD_zbornik/007-010_6ZD_zbornik-Primic_Zakelj-Ivanus.pdf (accessed 26 August 2022)
- Annual report 2013. https://zora.onko-i.si/fileadmin/user_upload/publikacije/izobrazevanja/2014_05ZD_zbornik/5zd_zbornik-p7-11-PRIMIC_ZAKELJ_in_sod.pdf (accessed 26 August 2022)
- Annual report 2012. https://zora.onko-i.si/fileadmin/user_upload/publikacije/izobrazevanja/2013_04ZD_zbornik/4ZD_zbornik_1_ZAKELJ_in_sod_web.pdf (accessed 26 August 2022)
- Annual report 2011. https://zora.onko-i.si/fileadmin/user_upload/publikacije/izobrazevanja/2012_03ZD_zbornik/TZD2012_11_ZAKELJ_in_sod_zbornik.pdf (accessed 26 August 2022)
- Annual report 2010. https://zora.onko-i.si/fileadmin/user_upload/publikacije/izobrazevanja/2011_02ZD_zbornik/2011_DZD_12_ZORA_2005-2009_web.pdf (accessed 26 August 2022)
- Annual report 2009. https://zora.onko-i.si/fileadmin/user_upload/publikacije/izobrazevanja/2010_01ZD_zbornik/1_DP_ZORA_DANES_PZD_2010_web.pdf (accessed 26 August 2022)
- Annual report 2007 and 2008. https://zora.onko-i.si/fileadmin/user_upload/publikacije/letna_porocila/letno_porocilo_2007_08.pdf (accessed 26 August 2022)
- Annual report 2006 and 2007. https://zora.onko-i.si/fileadmin/user_upload/publikacije/letna_porocila/letno_porocilo_2006_07.pdf (accessed 26 August 2022)
- Annual report 2004 and 2005. https://zora.onko-i.si/fileadmin/user_upload/publikacije/letna_porocila/letno_porocilo_2004_05.pdf (accessed 26 August 2022)
- Annual report 2003 and 2004. https://zora.onko-i.si/fileadmin/user_upload/publikacije/letna_porocila/letno_porocilo_2003_04.pdf (accessed 26 August 2022)

Spain

- Luces Lago AM, Mosquera Pan L, López Folgueiras B, Tizón Bouza E. Nuevo enfoque en el Programa de cribado para la detección precoz del cáncer de cérvix en Galicia [New approach in the Screening Program for the early detection of cervical cancer in Galicia.]. *Rev Esp Salud Publica*. 2021;95:e202110129. Published 2021 Oct 4.

Sweden

- Nationellt Kvalitetsregister för Cervixcancerprevention. https://www.nkcx.se/article_e.htm (accessed 26 August 2022)
- Prevention of cervical cancer in Sweden. Activity report and Annual report 2022. https://www.nkcx.se/templates/_rsrapport_2022.pdf (accessed 26 August 2022)
- Prevention of cervical cancer in Sweden. Activity report and Annual report 2021. https://www.nkcx.se/templates/_rsrapport_2021.pdf (accessed 26 August 2022)
- Prevention of cervical cancer in Sweden. Activity report and Annual report 2020. https://www.nkcx.se/templates/_rsrapport_2020.pdf (accessed 26 August 2022)
- Prevention of cervical cancer in Sweden. Activity report and Annual report 2019. https://www.nkcx.se/templates/_rsrapport_2019.pdf (accessed 26 August 2022)

- Prevention of cervical cancer in Sweden. Activity report and Annual report 2018. https://www.nkcx.se/templates/_rsrapport_2018.pdf (accessed 26 August 2022)
- Prevention of cervical cancer in Sweden. Activity report and Annual report 2017. https://www.nkcx.se/templates/_rsrapport_2017.pdf (accessed 26 August 2022)
- Prevention of cervical cancer in Sweden. Activity report and Annual report 2016. https://www.nkcx.se/templates/_rsrapport_2016.pdf (accessed 26 August 2022)
- Prevention of cervical cancer in Sweden. Activity report and Annual report 2015. https://www.nkcx.se/templates/_rsrapport_2015.pdf (accessed 26 August 2022)
- Prevention of cervical cancer in Sweden. Activity report and Annual report 2014. https://www.nkcx.se/templates/_rsrapport_2014.pdf (accessed 26 August 2022)
- Prevention of cervical cancer in Sweden. Activity report and Annual report 2013. https://www.nkcx.se/templates/_rsrapport_2013.pdf (accessed 26 August 2022)
- Prevention of cervical cancer in Sweden. Activity report and Annual report 2012. https://www.nkcx.se/templates/_rsrapport_2012.pdf (accessed 26 August 2022)
- Prevention of cervical cancer in Sweden. Activity report and Annual report 2011. https://www.nkcx.se/templates/_rsrapport_2011.pdf (accessed 26 August 2022)
- National evaluation cervical screening Method description. Appendix 3. https://www.socialstyrelsen.se/globalassets/sharepoint-dokument/artikelkatalog/nationella-riktlinjer/2020-6-6800-metodbilaga.pdf (accessed 26 August 2022)
- National evaluation cervical screening Method description Appendix 3 National evaluation cervical screening. Indicators Appendix 2. https://www.socialstyrelsen.se/globalassets/sharepoint-dokument/artikelkatalog/nationella-riktlinjer/2020-6-6800-indikatorer.pdf (accessed 26 August 2022)

Switzerland

- Catarino R, Vassilakos P, Petignat P, Combescure C. Harms and benefits of cervical cancer screening among non-attenders in Switzerland: The transition towards HPV-based screening. *Prev Med Rep*. 2022;29:101929. Published 2022 Jul 30. doi:10.1016/j.pmedr.2022.101929

Ukraine

- CERVICAL CANCER IN UKRAINE: THE CONTINUUM OF CARE AND IMPLICATIONS FOR ACTION. https://openknowledge.worldbank.org/bitstream/handle/10986/31156/133694-WP-PUBLIC-ADD-SERIES-CervicalCancerinUkrainePolicybriefENGLFINAL.pdf?sequence=1&isAllowed=y, last accessed: 27 August 2022)
- Kolesnyk, P., Frese, T., Vinker, S. *et al.* Steps towards implementing evidence-based screening in family medicine in Ukraine: SWOT-analysis of an approach of multidimensional empowerment. *BMC Fam Pract* **22**, 20 (2021). https://doi.org/10.1186/s12875-021-01367-2

UK – England

- Invasive cervical cancer audits. https://www.gov.uk/government/collections/cervical-screening-programme-data#invasive-cervical-cancer-audits (accessed 27 August 2022).
- Cervical screening: annual standards report. https://www.gov.uk/government/collections/cervical-screening-programme-data#cervical-screening:-annual-standards-report (accessed 27 August 2022).

- https://www.qmul.ac.uk/wolfson/centres/ccp/news/general-news/items/nhscsp-audit-of-invasive-cervicalcancer.html (accessed 27 August 2022).
- National invasive cervical cancer audit. Updated 29 September 2021. https://www.gov.uk/government/publications/cervical-screening-auditing-procedures/national-invasive-cervical-cancer-audit (accessed 27 August 2022).
- Cervical screening standards valid for data collected from 1 April 2020. https://www.gov.uk/government/publications/cervical-screening-programme-standards/cervical-screening-programme-standards-valid-for-data-collected-from-1-april-2018 (accessed 27 August 2022)
- Cervical screening: laboratories providing HPV testing and cytology services in the NHS Cervical Screening Programme. https://www.gov.uk/government/publications/cervical-screening-laboratory-hpv-testing-and-cytology-services/cervical-screening-guidance-for-laboratories-providing-hpv-testing-and-cytology-services-in-the-nhs-cervical-screening-programme (accessed 27 August 2022)

UK – Northern Ireland

- Reviewing your cervical screening history: information for women diagnosed with cervical cancer. https://www.publichealth.hscni.net/publications/reviewing-your-cervical-screening-history-information-women-diagnosed-cervical-cancer (accessed 27 August 2022)
- Audit of invasive cervical cancer protocol. . https://cancerscreening.hscni.net/download/27/cervical-resources-for-professionals/849/ni-protocol_audit-of-invasive-cervical-cancers_december-2014.pdf, last accessed 27 August 2022.
- Programme performance and standards. 2017-2021. https://cancerscreening.hscni.net/cervical-screening/programme-performance-and-standards/ (accessed 27 August 2022)

UK – Scotland

- Scottish cervical screening programme statistics publication. https://www.isdscotland.org/health-topics/cancer/cervical-screening/ (accessed 27 August 2022)
- Scottish cervical screening programme statistics. https://publichealthscotland.scot/publications/scottish-cervical-screening-programme-statistics/scottish-cervical-screening-programme-statistics-annual-update-to-31-march-2022/ (accessed 27 August 2022)

UK – Wales

- Cervical screening Wales. Annual statistics report 2019-20. https://phw.nhs.wales/services-and-teams/cervical-screening-wales/information-resources/programme-reports/csw-annual-statistical-reports/csw-annual-statistical-report-2019-20/ (accessed 27 August 2022)
- Cervical screening Wales. Annual statistics report 2018-19. https://phw.nhs.wales/services-and-teams/cervical-screening-wales/information-resources/programme-reports/csw-annual-statistical-reports/csw-annual-statistical-report-2018-19/ (accessed 27 August 2022)
- Cervical screening Wales. Annual statistics report 2016-17. https://phw.nhs.wales/services-and-teams/cervical-screening-wales/information-resources/programme-reports/csw-annual-statistical-reports/csw-annual-statistical-report-2016-17/ (accessed 27 August 2022)

Other references

- Cervix cancer screening/IARC Working Group on the Evaluation of Cancer Preventive Strategies (2004:Lyon, France) (IARC Handbooks of Cancer Prevention ;10). Table 73. https://publications.iarc.fr/_publications/media/download/3960/ff5840132ff86533aa8d965afba99a597070e8e8.pdf

- International Agency for Research on Cancer. European guidelines for quality assurance in cervical cancer screening. Second edition. 2008. https://screening.iarc.fr/doc/ND7007117ENC_002.pdf
- Ronco G, van Ballegooijen M, Becker N, et al. Process performance of cervical screening programmes in Europe. *Eur J Cancer*. 2009;45(15):2659-2670. doi:10.1016/j.ejca.2009.07.022

REFERENCES FOR COLORECTAL SCREENING PROGRAMMES

Albania

- No organized screening programme nor report.

Andorra

- No organized screening programme nor report.

Austria

- Colon cancer screening. https://eprints.aihta.at/983/1/HTA-Projektbericht_Nr.41c.pdf (accessed 19 April 2023)
- Brief overview of the preparation of the quality standard for screening colonoscopy. https://goeg.at/sites/goeg.at/files/inline-files/Kurz%C3%BCbersicht%20Vorbereitung%20QS%20Vorsorgekoloskopie_FG%20VP_28-06-2022.pdf (accessed 19 April 2023)
- Allen JI. Quality assurance for gastrointestinal endoscopy. *Curr Opin Gastroenterol*. 2012;28(5):442-450. doi:10.1097/MOG.0b013e3283561f0d
- Kaminski MF, Regula J, Kraszewska E, et al. Quality indicators for colonoscopy and the risk of interval cancer. *N Engl J Med*. 2010;362(19):1795-1803. doi:10.1056/NEJMoa0907667

Belarus

- No organized screening programme nor report.

Belgium

- Colorectal cancer. http://kankerregister.org/Colorectal_Cancer (accessed 19 April 2023)
- Annual Reports 2022. Colorectal cancer. https://dikkedarmkanker.bevolkingsonderzoek.be/nl/ddk/literatuur-ddk (accessed 14 March 2023)
- Sanduleanu S, le Clercq CMC, Dekker E On behalf of the Expert Working Group on '*Right-sided lesions* and *interval cancers*', Colorectal Cancer Screening Committee, World Endoscopy Organization, *et al*. Definition and taxonomy of interval colorectal cancers: a proposal for standardising nomenclature. *Gut* 2015;64:1257-1267
- Annual reports 2014-2022. https://dikkedarmkanker.bevolkingsonderzoek.be/nl/ddk/literatuur-ddk (accessed 14 March 2023)
- Informations sur le Programme de dépistage du cancer colorectal en Fédération Wallonie-Bruxelles. https://www.ccref.org/pro/pdf/publications/feedback_colon_2015.pdf (accessed 14 March 2023)
- van de Veerdonk W, Hoeck S, Peeters M, Van Hal G, Francart J, De Brabander I. Occurrence and characteristics of faecal immunochemical screen-detected cancers vs non-screen-detected cancers: Results from a Flemish colorectal cancer screening programme. *United European Gastroenterol J*. 2020;8(2):185-194. doi:10.1177/2050640619882157

Bosnia and Herzegovina

- No organized screening programme nor report.

Bulgaria

- No organized screening programme nor report.

Croatia

- Katičić M, Antoljak N, Kujundžić M, et al. Results of National Colorectal Cancer Screening Program in Croatia (2007-2011). *World J Gastroenterol*. 2012;18(32):4300-4307. doi:10.3748/wjg.v18.i32.4300
- NATIONAL PROGRAM FOR EARLY DETECTION OF COLON CANCER. https://zdravlje.gov.hr/UserDocsImages/dokumenti/Programi,%20projekti%20i%20strategije/Nacionalni%20program%20ranog%20otkrivanja%20raka%20debelog%20crijeva.pdf (accessed 19 April 2023)
- National Colorectal Cancer Early Detection Program.https://www.zzjzdnz.hr/projekti/nacionalni-program-ranog-otkrivanja-raka-debelog-crijeva (accessed 19 April 2023)

Cyprus

- The National Summary of cancer plan of Cyprus. http://www.epaac.eu/from_heidi_wiki/Cyprus_Summary_of_NCP_English.pdf (accessed 19 April 2023)
- Free screenings for bowel cancer. https://cyprus-mail.com/2022/03/22/free-screenings-for-bowel-cancer/ (accessed 19 April 2023)

Czechia

- SCREENING PROGRAMME IN THE CZECH REPUBLIC. https://www.registry.cz/index-en.php?pg=registries&prid=51 (accessed 19 April 2023)
- Colorectal cancer screening. https://www.kolorektum.cz/en/ (accessed 19 April 2023)
- Suchanek, Stepana; Majek, Ondreje; Vojtechova, Gabrielaaet al. Colorectal cancer prevention in the Czech Republic: time trends in performance indicators and current situation after 10 years of screening. European Journal of Cancer Prevention 23(1):p 18-26, January 2014. | DOI: 10.1097/CEJ.0b013e328364f203
- Zavoral M, Suchanek S, Majek O, et al. Colorectal cancer screening: 20 years of development and recent progress. *World J Gastroenterol*. 2014;20(14):3825-3834. doi:10.3748/wjg.v20.i14.3825

Denmark

- Danish Bowel Cancer Screening Database Annual Report 2021: https://www.sundhed.dk/content/cms/45/61245_dts_aarsrapport2021_til-offentliggrelse_13122022.pdf (accessed 19 April 2023)
- Indicators and standards for the Danish Bowel Cancer Screening Database. https://www.rkkp-dokumentation.dk/Public/Pdf/Default.aspx?db=63&report=2&filetype=pdf&db2=1000000506 (accessed 19 April 2023)
- Danish Bowel Cancer Screening Database Annual Report 2020. https://www.sundhed.dk/content/cms/45/61245_dts_aarsrapport_2020_endelig-version_10122021.pdf (accessed 19 April 2023)

- Danish Bowel Cancer Screening Database Annual Report 2019. https://www.sundhed.dk/content/cms/45/61245_aarsrapport2019_dts_til-offentliggoerelse_16032021.pdf (accessed 19 April 2023)
- Danish Bowel Cancer Screening Database Annual Report 2018. https://www.rkkp.dk/siteassets/forside/databaser/arsrapporter/dts-arsrapport2018_anonymiseret_endelig_marts2020.pdf (accessed 19 April 2023)
- Danish Bowel Cancer Screening Database Annual Report 2017. https://www.sundhed.dk/content/cms/45/61245_dts_%C3%A5rsrapport-2017_final.pdf (accessed 19 April 2023)
- Danish Bowel Cancer Screening Database Annual Report 2016, https://www.regionshospitalet-randers.dk/siteassets/afdelinger/afdeling-for-folkeundersogelser/pdf-episerver/retningslinjer/20171221_dtsarsrapport-2016_offentlig-version.pdf (accessed 19 April 2023)

Estonia

- Colon cancer screening. https://www.haigekassa.ee/en/people/health-prevention/colon-cancer-screening (accessed 19 April 2023)
- Estonian Cancer Screening Registry. https://en.tai.ee/en/r-and-d/registers/estonian-cancer-screening-registry (accessed 19 April 2023)
- Cancer screening programmes database. https://statistika.tai.ee/pxweb/en/Andmebaas/Andmebaas__02Haigestumus__07Soeluuringud/?tablelist=true (accessed 19 April 2023)

Finland

- Colorectal cancer screening. https://cancerregistry.fi/research/research-themes/colorectal-cancer-screening/ (accessed 19 April 2023)
- A GUIDE TO ORGANIZING BOWEL CANCER SCREENING. https://syoparekisteri.fi/assets/files/2021/09/Opas-suolistosyovan-seulonnan-jarjestamiseksi.pdf. (accessed 19 April 2023)
- Rex DK, Schoenfeld PS, Cohen J, et al. Quality indicators for colonoscopy. *Am J Gastroenterol*. 2015;110(1):72-90. doi:10.1038/ajg.2014.385
- Colorectal cancer screening. https://stats.cancerregistry.fi/joukkistilastot/2013-2014/suolisto.html. (accessed 19 April 2023)

France

- The National Colorectal Cancer Screening Program. https://www.e-cancer.fr/Professionnels-de-sante/Depistage-et-detection-precoce/Depistage-du-cancer-colorectal/Le-programme-national-de-depistage (accessed 19 April 2023)
- SCREENING FOR COLORECTAL CANCER. Organized national screening program. https://www.e-cancer.fr/content/download/444160/6715797/file/Diaporama%20national%20de%20pr%C3%A9sentation%20du%20programme%20de%20d%C3%A9pistage%20organis%C3%A9%20du%20cancer%20colorectal%20%E2%80%93%2009-2022.pdf (accessed 19 April 2023)
- MEDICO-ECONOMIC EVALUATION OF COLORECTAL CANCER SCREENING / Technical report. https://www.e-cancer.fr/content/download/263559/3710578/file/Evaluation_medico_eco_DOCCR_rapport_technique_mel_20190517.pdf (accessed 19 April 2023)

- MINISTRY OF SOLIDARITY AND HEALTH. https://www.e-cancer.fr/content/download/429186/6466374/file/Programme%20de%20d%C3%A9pistage%20organis%C3%A9%20du%20cancer%20colorectal%20-%20arr%C3%AAt%C3%A9%20du%2001-04-2022.pdf (accessed 19 April 2023)
- Evaluation of the organized screening program for colon-rectal cancer over the period 2018-2019 and 2020: national indicators. https://www.santepubliquefrance.fr/maladies-et-traumatismes/cancers/cancer-du-colon-rectum/articles/evaluation-du-programme-de-depistage-organise-du-cancer-du-colon-rectum-sur-la-periode-2018-2019-et-2020-indicateurs-nationaux (accessed 19 April 2023)
- National organized screening program for colorectal cancer - Evaluation over the period 2020. https://www.santepubliquefrance.fr/media/files/01-maladies-et-traumatismes/cancers/cancer-du-colon-rectum/depistage-organise-du-cancer-colorectal-guide-du-format-des-donnees-et-definitions-des-indicateurs-de-l-evaluation-du-programme-national (accessed 19 April 2023)
- National organized screening program for colorectal cancer - Evaluation over the period 2018-2019. https://www.santepubliquefrance.fr/media/files/01-maladies-et-traumatismes/cancers/cancer-du-colon-rectum/evaluation-du-programme-de-depistage/periode-2018-2019-2020/national/tableau3_2018_2019-pdf (accessed 19 April 2023)
- Giai J, Exbrayat C, Boussat B, et al. Sensitivity of a colorectal cancer screening program based on a guaiac test: a population-based study. *Clin Res Hepatol Gastroenterol*. 2014;38(1):106-111. doi:10.1016/j.clinre.2013.06.011

Germany

- New task, new data. https://www.krebsdaten.de/Krebs/EN/Home/teaser_news/Teaser_legal_changes.html (accessed 19 April 2023)
- Colorectal cancer - early detection. https://www.krebsgesellschaft.de/onko-internetportal/basis-informationen-krebs/krebsarten/darmkrebs/frueherkennung.html: (accessed 19 April 2023)
- Colorectal cancer screening program. https://www.g-ba.de/themen/methodenbewertung/ambulant/frueherkennung-krankheiten/erwachsene/krebsfrueherkennung/darmkrebs-screening (accessed 19 April 2023)
- Joint Federal Committee for Organized Early Cancer Detection Programs. https://www.g-ba.de/downloads/62-492-3039/oKFE-RL-2022-11-17-iK-2023-01-26.pdf (accessed 19 April 2023)

Greece

- COLORECTAL SCREENING ACROSS EUROPE. https://ueg.eu/files/779/67d96d458abdef21792e6d8e590244e7.pdf (accessed 19 April 2023)
- J.K. Triantafillidis, Paris A. Kosmidis, A et al. Screening programs for colorectal cancer in Greece: Results of two pilot studies conducted in March 2008 and 2009. Annals of Gastroenterology. Volume 23, No 1 (2010) https://digestiveoncology.org.gr/wp-content/uploads/2019/03/SCREENING-FOR-CRC-IN-GREECE.pdf

Hungary

- COLORECTAL SCREENING. https://egeszsegvonal.gov.hu/en/stay-healthy/colorectal-screening.html (accessed 19 April 2023)
- Rutka M, Bor R, Molnár T, et al. Efficacy of the population-based pilot colorectal cancer screening, Csongrád county, Hungary, 2015. *Turk J Med Sci*. 2020;50(4):756-763. Published 2020 Jun 23. doi:10.3906/sag-1908-79

Iceland

- What are the benefits of colorectal cancer screening? https://www.krabb.is/krabbameinsleit/ristilkrabbameinsleit/ (accessed 19 April 2023)

Ireland

- BowelScreen Programme Report 2018 – 2019 Round Three. https://www.bowelscreen.ie/_fileupload/Programme%20Reports/BowelScreen%20Programme%20Report%20Round%20Three.pdf. (accessed 19 April 2023)
- BowelScreen Programme Report 2016 – 2017 Round Two. https://www.bowelscreen.ie/_fileupload/Programme%20Reports/BowelScreen-Programme-Report%20-2016-2017-FINAL-WEB-21_01_20.pdf (accessed 19 April 2023)
- Interval Cancer Report BowelScreen. October 2020. https://assets.hse.ie/media/documents/BowelScreen_Interval_Cancer_Report_2020.pdf (accessed 19 April 2023)
- Guidelines for Quality Assurance in Colorectal Screening. Second Edition. https://www.screeningservice.ie/publications/BS-Guidelines-for-Quality-Assurance-in-Colorectal-Screening.pdf (accessed 19 April 2023)

Italy

- Colorectal screening. Activity data from 20018 to 2021. https://www.osservatorionazionalescreening.it/content/lo-screening-colorettale (accessed 19 April 2023)
- COLORECTAL SCREENING SEEN BY "PASSI". https://www.osservatorionazionalescreening.it/content/lo-screening-colorettale-visto-da-passi (accessed 19 April 2023)
- National screening observatory Report on 2019. https://www.osservatorionazionalescreening.it/sites/default/files/allegati/Rapportosul2019.pdf (accessed 19 April 2023)
- National screening observatory Report on 2020. https://www.osservatorionazionalescreening.it/sites/default/files/allegati/Rapporto%20ONS%202020_Definitivo_0.pdf (accessed 19 April 2023)
- National screening observatory Report on 2017. https://www.osservatorionazionalescreening.it/sites/default/files/allegati/ons%20rapporto%202017.pdf (accessed 19 April 2023)

Latvia

- Organised colorectal cancer screening needed in Latvia. https://cancercontrol.eu/archived/news/10/26/Organised-colorectal-cancer-screening-needed-in-Latvia/d%2Cnews.html (accessed 19 April 2023)

- Colorectal cancer in Latvia - diagnosis, treatment options and problems. http://www.europacolon-latvia.lv/uploads/files/ppp_dr_plate_latvia.pdf (accessed 19 April 2023)

Lithuania

- Dulskas A, Poskus T, Kildusiene I, et al. National Colorectal Cancer Screening Program in Lithuania: Description of the 5-Year Performance on Population Level. *Cancers (Basel)*. 2021;13(5):1129. Published 2021 Mar 6. doi:10.3390/cancers13051129

Luxembourg

- The White Paper on Colorectal Cancer in Luxembourg. https://plancancer.files.wordpress.com/2014/08/livre-blanc-cancer-colorectal-luxembourg1.pdf (accessed 19 April 2023)

Malta

- Colorectal screening. https://deputyprimeminister.gov.mt/en/phc/nbs/Pages/Screening-Programmes/Colorectal-Screening.aspx (accessed 19 April 2023)
- Annual reports for primary healthcare during 2018-2021. https://deputyprimeminister.gov.mt/en/phc/Pages/Publications/Annual-Reports.aspx (accessed 19 April 2023)
- Malta Colorectal Cancer Awareness Group. https://www.maltahealthnetwork.org/wp-content/uploads/2021/01/MCRCAG-Presentation.pdf (accessed 19 April 2023)

Monaco

- Mars Bleu: colorectal cancer awareness month Over 50s – get tested in 5 minutes. https://en.gouv.mc/Policy-Practice/Social-Affairs-and-Health/News/Mars-Bleu-colorectal-cancer-awareness-month-Over-50s-get-tested-in-5-minutes (accessed 19 April 2023)

Montenegro

- Strengthening cancer screening in Montenegro. https://www.integratedcare4people.org/media/files/Montenegro.pdf (accessed 19 April 2023)

The Netherlands

- Colorectal cancer screening programme. https://www.rivm.nl/en/colorectal-cancer-screening-programme (accessed 19 April 2023)
- Monitoring report 2014-2021: https://www.rivm.nl/en/national-monitoring-of-colorectal-cancer-screening-programme (accessed 19 April 2023)
- Evaluation of the Colorectal Cancer Screening Programme 2018-2021. https://www.rivm.nl/en/documenten/evaluation-of-colorectal-cancer-screening-programme-2018-2021 (accessed 19 April 2023)
- National monitoring colorectal cancer screening programme 2021. https://www.rivm.nl/en/documenten/monitor-colorectal-cancer-2021 (accessed 19 April 2023)
- National monitoring colorectal cancer screening programme 2020. https://www.rivm.nl/en/media/199021 (accessed 19 April 2023)
- National monitoring colorectal cancer screening programme 2019. https://www.rivm.nl/en/media/169811 (accessed 19 April 2023)

- National monitoring colorectal cancer screening programme 2018. https://www.rivm.nl/en/documenten/monitoring-of-colorectal-cancer-screening-programme-2018 (accessed 19 April 2023)
- National monitoring colorectal cancer screening programme 2017. https://www.rivm.nl/en/media/100661 (accessed 19 April 2023)
- National monitoring colorectal cancer screening programme 2016. https://www.rivm.nl/en/media/85051 (accessed 19 April 2023)
- National monitoring colorectal cancer screening programme 2015. https://www.rivm.nl/en/media/70681 (accessed 19 April 2023)
- National monitoring colorectal cancer screening programme 2014. https://www.rivm.nl/en/media/77811 (accessed 19 April 2023)

North Macedonia

- Colorectal Cancer Awareness Month -2019. https://www.iph.mk/en/colorectal-cancer-awareness-month-2019/ (accessed 19 April 2023)

Norway

- Promising results for colon cancer screening. https://sciencenorway.no/cancer-colon-cancer-screening/promising-results-for-colon-cancer-screening/1803090 (accessed 19 April 2023)
- Colorectal Cancer Screening. https://www.kreftregisteret.no/en/screening/Screening-for-colorectal-cancer/ (accessed 19 April 2023)
- The bowel screening programme's quality manual. https://www.kreftregisteret.no/screening/tarmscreening/for-helsepersonell/kvalitetsmanual/ (accessed 19 April 2023)
- The bowel screening programme's quality manual. Chapter 12. Monitoring and quality assurance. https://www.kreftregisteret.no/globalassets/tarmkreftscreening/dokumenter/kvalitetsmanualen/kapittel-12.-monitorering-og-kvalitetssikring.pdf. (accessed 19 April 2023)

Poland

- Colorectal cancer screening programme in Poland – pathologist's persepctives. https://docs.kmcongress.com/iaphd2017/slides/a-mroz.pdf (accessed 19 April 2023)
- Colorectal cancer. http://www.puo.pl/badania-profilaktyczne/rak-jelita-grubego. (accessed 19 April 2023)
- Bugajski M, Rupinski M, Wieszczy P, Pisera M, Regula J, Kaminski MF. Key performance measures for colonoscopy in the Polish Colonoscopy Screening Program. *Endoscopy*. 2019;51(9):858-865. doi:10.1055/a-0956-1889

Portugal

- Khan H, Shaaban N, Peleteiro B. Faecal occult blood test and colonoscopy use in Portugal: Results from the National Health Survey 2014. *J Med Screen*. 2020;27(4):171-185. doi:10.1177/0969141319891456
- Programa de Rastreio de Cancro Colo-Rectal no Norte de Portugal: Primeiros Hugo MONTEIRO, Fernando TAVARES, João REIS, et al. Acta Med Port 2022 Mar;35(3):164-169. https://www.actamedicaportuguesa.com/revista/index.php/amp/article/view/15904/6398 (accessed 19 April 2023)

Republic of Moldova

- No organized screening programme nor report.

Romania

- The first Romanian pilot project for screening colorectal cancer $4,000. https://www.romanianunitedfund.org/_the_first_romanian_pilot_project_for_screening_colorectal_cancer (accessed 19 April 2023)

Russian Federation

- Colorectal cancer screening in the regions of the Russian Federation. https://www.worldendo.org/wp-content/uploads/2018/11/181019_CRC-SC-Vienna_S-Konovalov.pdf (accessed 19 April 2023)
- Colorectal cancer screening: global situation and recommended colonoscopy quality standards. https://www.mediasphera.ru/issues/dokazatelnaya-gastroenterologiya/2017/4/downloads/ru/1230522602017041032 (accessed 19 April 2023)

San Marino

- No organized screening programme nor report.

Serbia

- Banković Lazarević D, Krivokapić Z, Barišić G, Jovanović V, Ilić D, Veljković M. Organized colorectal cancer screening in Serbia - the first round within 2013-2014. *Vojnosanit Pregl*. 2016;73(4):360-367. doi:10.2298/VSP150421113B. https://pubmed.ncbi.nlm.nih.gov/29309104/
- REGULATION ON THE NATIONAL PROGRAM FOR EARLY DETECTION OF COLORECTAL CANCER. https://www.skriningsrbija.rs/files/File/English/REGULATION_ON_THE_NATIONAL_PROGRAM_FOR_EARLY_DETECTION_OF_COLORECTAL_CANCER.pdf (accessed 19 April 2023)
- Colorectal cancer screening. https://www.skriningsrbija.rs/eng/colorectal-cancer-screening/ (accessed 19 April 2023)

Slovakia

- Cancer Screening Programs in Slovakia. Report 2020. https://www.noisk.sk/files/2021/2021-08-26-noi-rocenka-skriningy-2020-en.pdf (accessed 19 April 2023)
- Preliminary evaluation of the 1st phase of population screening for colorectal cancer. https://www.noisk.sk/files/2020/2020-11-12-predbezne-vysledky-prvej-fazy-skriningu-kolorektalneho-karcinomu.pdf (accessed 19 April 2023)

Slovenia

- Svit Programme. https://www.program-svit.si/ (accessed 19 April 2023)
- http://www.dpor.si/eng/wp-content/uploads/2018/11/DPOR_POROCILO2018_SVIT_ang_posredovano.pdf. (accessed 19 April 2023)
- Slovenian guidelines for ensuring the quality of colon and rectal cancer screening. https://www.program-svit.si/wp-content/uploads/2019/01/SmerniceProgramaSvit.pdf (accessed 19 April 2023)

- Tepes B, Stefanovic M, Stabuc B, Mlakar DN, Grazio SF, Zakotnik JM. Quality Control in the Slovenian National Colorectal Cancer Screening Program. *Dig Dis*. 2022;40(2):187-197. doi:10.1159/000516978 https://www.karger.com/Article/FullText/516978#ref5
- SLOVENIA – AN EARLY AND SUCCESSFULLY ORGANISED POPULATION-BASED SCREENING PROGRAMME. https://www.worldendo.org/wp-content/uploads/2016/04/Program-Svit-WEO-Barcelona-2019.pdf (accessed 19 April 2023)

Spain

- COLORECTAL CANCER SCREENING PROGRAM. https://www.osakidetza.euskadi.eus/contenidos/informacion/deteccion_cancer_colorrectal/es_def/adjuntos/PROGRAMA_DEFINITIVO_22022021.pdf (accessed 19 April 2023)
- COLORECTAL CANCER SCREENING PROGRAM. https://www.osakidetza.euskadi.eus/enfermedad-cancer/-/programa-cribado-cancer-colorrectal/. (accessed 19 April 2023)
- https://www.osakidetza.euskadi.eus/contenidos/informacion/deteccion_cancer_colorrectal/es_def/adjuntos/GENERALES_22022021.pdf (accessed 19 April 2023)
- Salas Trejo, Doloresa; Portillo Villares, Isabeld; Espinàs Piñol et al. Spanish Cancer Screening Network. Implementation of colorectal cancer screening in Spain: main results 2006–2011. European Journal of Cancer Prevention 26(1):p 17-26, January 2017. | DOI: 10.1097/CEJ.0000000000000232 https://journals.lww.com/eurjcancerprev/Abstract/2017/01000/Implementation_of_colorectal_cancer_screening_in.3.aspx
- Binefa G, Garcia M, Milà N, et al. Colorectal Cancer Screening Programme in Spain: Results of Key Performance Indicators After Five Rounds (2000-2012). *Sci Rep*. 2016;6:19532. Published 2016 Jan 20. doi:10.1038/srep19532 https://www.ncbi.nlm.nih.gov/pmc/articles/PMC4726304/pdf/srep19532.pdf.

Sweden

- Screening for colon and rectal cancer. Recommendation and assessment basis. https://www.socialstyrelsen.se/globalassets/sharepoint-dokument/artikelkatalog/nationella-screeningprogram/2014-2-31.pdf (accessed 19 April 2023)
- Colon and rectal cancer screening. Indicators. https://www.socialstyrelsen.se/globalassets/sharepoint-dokument/artikelkatalog/nationella-screeningprogram/screening-tjockandtarmscancer-indikatorer.pdf (accessed 19 April 2023)

Switzerland

- Cancer screening programs in your area. https://www.swisscancerscreening.ch/angebote-in-ihrem-kanton (accessed 19 April 2023)
- Swiss cancer screening 2021. https://www.swisscancerscreening.ch/fileadmin/user_upload/scs_Gescha__ftsbericht_DE_2021_220518.pdf (accessed 19 April 2023)

Ukraine

- Welten VM, Wanis KN, Semeniv S, et al. Colonoscopy Needs for Implementation of a Colorectal Cancer Screening Program in Ukraine. *World J Surg*. 2022;46(10):2476-2486. doi:10.1007/s00268-022-06656-0 https://pubmed.ncbi.nlm.nih.gov/35835863/

UK – England

- "Bowel cancer screening programme standards: valid for data collected from 1 April 2018. https://www.gov.uk/government/publications/bowel-cancer-screening-programme-standards/bowel-cancer-screening-programme-standards-valid-for-data-collected-from-1-april-2018#bcsp-s12-interventiontreatment-adenoma-detection-rate-fobt. (accessed 19 April 2023)
- Bowel cancer screening programme supporting information. https://www.gov.uk/government/publications/bowel-cancer-screening-programme-standards/our-approach-to-bowel-cancer-screening-standards (accessed 19 April 2023)

UK – Northern Ireland

- Bowel cancer screening programme. https://www.nidirect.gov.uk/bowel-screening#:~:text=The%20bowel%20cancer%20screening%20programme%20is%20for%20people%20who%20have,do%20the%20test%20at%20home. (accessed 19 April 2023)
- Programme Performance and Standards. https://cancerscreening.hscni.net/bowel-screening/programme-performance-and-standards/. (accessed 19 April 2023)
- Bowel cancer screening programme standards: valid for data collected from 1 April 2018. https://www.gov.uk/government/publications/bowel-cancer-screening-programme-standards/bowel-cancer-screening-programme-standards-valid-for-data-collected-from-1-april-2018 (accessed 19 April 2023)

UK – Scotland

- Scottish Bowel Screening Programme. https://www.isdscotland.org/health-topics/cancer/bowel-screening/ (accessed 19 April 2023)
- Scottish Bowel Screening Programme. Key Performance Indicators Report: May 2019 data submission. Invitations between 1st November 2016 and 31st October 2018. https://view.officeapps.live.com/op/view.aspx?src=https%3A%2F%2Fwww.isdscotland.org%2FHealth-Topics%2FCancer%2FPublications%2F2019-08-06%2F2019-08-06-Bowel-Screening-KPI-Report.xlsx&wdOrigin=BROWSELINK (accessed 19 April 2023)
- Scottish Bowel Screening Programme Statistics. For the two-year period of invitations between 1 November 2016 and 31 October 2018. https://www.isdscotland.org/Health-Topics/Cancer/Publications/2019-08-06/2019-08-06-Bowel-Screening-Publication-Report.pdf (accessed 19 April 2023)
- Scottish bowel screening programme statistics. For the period of invitations from May 2020 to April 2022. https://publichealthscotland.scot/media/17799/2023-02-21-bowel-screening-publication-report.pdf (accessed 19 April 2023)
- Scottish Bowel Screening Programme. Key Performance Indicators Report: Nov 2022 data submission. Invitations between 1 May 2020 and 30 April 2022. https://publichealthscotland.scot/media/17689/2023-02-21-bowel-screening-kpi-report.xlsx (accessed 19 April 2023)

UK – Wales

- Bowel screening Wales Annual Statistical Report 2019-20. https://phw.nhs.wales/services-and-teams/screening/bowel-screening/programme-reports/bsw-annual-statistical-reports/bsw-annual-statistical-report-2019-2020/
- Quality Assurance Reference Manual. https://phw.nhs.wales/services-and-teams/screening/bowel-screening/information-resources-old/bsw-quality-manual-version-01d/f (accessed 19 April 2023)

- BSW Annual Statistical Reports. https://phw.nhs.wales/services-and-teams/screening/bowel-screening/programme-reports/bsw-annual-statistical-reports/ (accessed 19 April 2023)
- BSW Annual Statistical Reports 2019-2020. https://phw.nhs.wales/services-and-teams/screening/bowel-screening/programme-reports/bsw-annual-statistical-reports/bsw-annual-statistical-report-2019-2020/ (accessed 19 April 2023)
- BSW Annual Statistical Reports 2018-2019. https://phw.nhs.wales/services-and-teams/screening/bowel-screening/programme-reports/bsw-annual-statistical-reports/bsw-annual-statistical-report-2018-19/ (accessed 19 April 2023)
- BSW Annual Statistical Reports 2017-2018. https://phw.nhs.wales/services-and-teams/screening/bowel-screening/programme-reports/bsw-annual-statistical-reports/bsw-annual-statistical-report-2017-18/ (accessed 19 April 2023)
- BSW Annual Statistical Reports 2016-2017. https://phw.nhs.wales/services-and-teams/screening/bowel-screening/programme-reports/bsw-annual-statistical-reports/bsw-annual-statistical-report-2016-17/ (accessed 19 April 2023)
- BSW Annual Statistical Reports 2015-2016. https://phw.nhs.wales/services-and-teams/screening/bowel-screening/programme-reports/bsw-annual-statistical-reports/bsw-annual-statistical-report-2015-16/ (accessed 19 April 2023)
- BSW Annual Statistical Reports 2014-2015. https://phw.nhs.wales/services-and-teams/screening/bowel-screening/programme-reports/bsw-annual-statistical-reports/bsw-annual-statistical-report-2014-15/ (accessed 19 April 2023)
- BSW Annual Statistical Reports 2013-2014. https://phw.nhs.wales/services-and-teams/screening/bowel-screening/programme-reports/bsw-annual-statistical-reports/bsw-annual-statistical-report-2013-14/ (accessed 19 April 2023)

Other references

- Moss S, Ancelle-Park R, Brenner H; International Agency for Research on Cancer. European guidelines for quality assurance in colorectal cancer screening and diagnosis. First Edition--Evaluation and interpretation of screening outcomes. *Endoscopy*. 2012;44 Suppl 3:SE49-SE64. doi:10.1055/s-0032-1309788. https://www.thieme-connect.com/products/ejournals/html/10.1055/s-0032-1309788
- Sanduleanu S, le Clercq CM, Dekker E, et al. Definition and taxonomy of interval colorectal cancers: a proposal for standardising nomenclature. *Gut*. 2015;64(8):1257-1267. doi:10.1136/gutjnl-2014-307992 https://gut.bmj.com/content/64/8/1257.long
